# Contrasting seasonality in optical-biogeochemical properties of the Baltic Sea

**DOI:** 10.1371/journal.pone.0173357

**Published:** 2017-04-06

**Authors:** Stefan G. H. Simis, Pasi Ylöstalo, Kari Y. Kallio, Kristian Spilling, Tiit Kutser

**Affiliations:** 1 Plymouth Marine Laboratory, Plymouth, United Kingdom; 2 Finnish Environment Institute SYKE, Marine Research Centre, Helsinki, Finland; 3 Estonian Marine Institute, University of Tartu, Tallinn, Estonia; University of Siena, ITALY

## Abstract

Optical-biogeochemical relationships of particulate and dissolved organic matter are presented in support of remote sensing of the Baltic Sea pelagic. This system exhibits strong seasonality in phytoplankton community composition and wide gradients of chromophoric dissolved organic matter (CDOM), properties which are poorly handled by existing remote sensing algorithms. Absorption and scattering properties of particulate matter reflected the seasonality in biological (phytoplankton succession) and physical (thermal stratification) processes. Inherent optical properties showed much wider variability when normalized to the chlorophyll-a concentration compared to normalization to either total suspended matter dry weight or particulate organic carbon. The particle population had the largest optical variability in summer and was dominated by organic matter in both seasons. The geographic variability of CDOM and relationships with dissolved organic carbon (DOC) are also presented. CDOM dominated light absorption at blue wavelengths, contributing 81% (median) of the absorption by all water constituents at 400 nm and 63% at 442 nm. Consequentially, 90% of water-leaving radiance at 412 nm originated from a layer (*z*_90_) no deeper than approximately 1.0 m. With water increasingly attenuating light at longer wavelengths, a green peak in light penetration and reflectance is always present in these waters, with *z*_90_ up to 3.0–3.5 m depth, whereas *z*_90_ only exceeds 5 m at biomass < 5 mg Chla m^-3^. High absorption combined with a weakly scattering particle population (despite median phytoplankton biomass of 14.1 and 4.3 mg Chla m^-3^ in spring and summer samples, respectively), characterize this sea as a dark water body for which dedicated or exceptionally robust remote sensing techniques are required. Seasonal and regional optical-biogeochemical models, data distributions, and an extensive set of simulated remote-sensing reflectance spectra for testing of remote sensing algorithms are provided as supplementary data.

## Introduction

The Baltic Sea is a brackish, semi-enclosed, and relatively shallow (average depth approximately 55 m) coastal sea with large latitudinal gradients in salinity and dissolved organic matter (DOM). Seasonality of the physics and hydrodynamics of the sea are driven by a permanent halocline, winter ice cover in northern sea areas and thermal stratification in summer [1]. Several large watersheds discharge into the sea while exchange with the open ocean is limited through the Danish Straits in the southwest. This limited water exchange, combined with high nutrient loads despite ongoing reduction efforts (HELCOM 2009, HELCOM 2013a), negatively affect water quality as evidenced by seasonal high-biomass phytoplankton blooms and extensive low oxygen zones. The diverse and seasonally variable conditions of the Baltic Sea thus create an interesting testing ground for novel environmental monitoring methods.

Remote sensing and in situ platforms, notably the ship-of-opportunity network *Alg@line*, are the dominant source of daily observations of the physical and biological state of the Baltic Sea. Optical in situ and remote observations provide particularly useful information on dynamics at the bottom of the food web. Generic ocean colour algorithms which extract information on phytoplankton pigment concentration from blue-to-green satellite waveband ratios are unsuitable for the Baltic Sea [2,3] due to high concentrations of chromophoric dissolved organic matter (CDOM) and high phytoplankton biomass during productive periods. Algorithms designed specifically to deal with the optical complexity of coastal waters have shown better performance separating CDOM, chlorophyll-*a* (Chla) and total suspended matter (TSM) concentrations but have thus far still required regional recalibration [4].

High concentrations of CDOM optically separate the Baltic Sea from other coastal seas. Absorption by CDOM at 412 nm (*a*_CDOM_(412), see Table 1 for a list of symbols and acronyms) can account for > 90% of total absorption in northern sea areas during clear water periods in summer [5] and 38–70% in the area influenced by the river Oder in the south in autumn [6]. Spatiotemporal variability of CDOM follows the major water masses with a dilution gradient from north to south [7–9]. First-order variability in *a*_CDOM_ therefore relates to seasonal ice melt and rainfall enhancing contributions from DOM-rich river sources in the Gulf of Bothnia and the Gulf of Finland in the north [10,11]. Second-order variability is driven by autochthonous DOM production during phytoplankton blooms [12], and photodegradation [13], both processes with strong seasonal drivers. Spatial variability is further enhanced by differences in DOM composition between catchments [14,15].

**Table 1:** Symbols and acronyms.

Acronym or Symbol	Description	Units
λ	Wavelength or waveband	nm
NIR	Near infra-red (> 700 nm)	
UV	Ultraviolet (< 400 nm)	
OLCI	Ocean and Land Colour Instrument	
**Sea areas (from north to south)**		
BoB	Bothnian Bay	
Qua	The Quark	
BoS	Bothnian Sea	
ÅlS	Aland Sea and Archipelago Sea	
GoF	Gulf of Finland	
NBP	Northern Baltic Proper	
WGB	Western Gotland Basin	
EGB	Eastern Gotland Basin	
BhB	Bornholm Basin	
ArB	Arkona Basin	
**Biogeochemical parameters**		
CDOM	Chromophoric Dissolved Organic Matter (<0.2 μm)	
Chla	(concentration of) Chlorophyll *a*	mg m^-3^
TSM	(concentration of) Total suspended matter	g m^-3^
ISM	(concentration of) Inorganic suspended matter	g m^-3^
POC	(concentration of) Particulate Organic Carbon	μM
PON	(concentration of) Particulate Organic Nitrogen	μM
POP	(concentration of) Particulate Organic Phosphorus	μM
**IOP**	**Inherent optical properties**	
*a*_x_(λ)	Absorption coefficient at wavelength or waveband λ, where subscript x is either (w) water, (p) particles > 0.7 μm, (ϕ) phytoplankton, (nap) ‘non-algal’ particles (nap = p—ϕ), or CDOM	m^-1^
*b*_x_(λ)	Scattering coefficient (see *a*_x_(λ) subscripts)	m^-1^
*b*_*b*x_(λ)	Backscattering coefficient (see *a*_x_(λ) subscripts)	m^-1^
*β*_p_(λ)	Particulate backcattering-to-scattering ratio at waveband λ (spectral average when λ is omitted)	dimensionless
*S*	Spectral slope coefficient of *a*_CDOM_(λ) model (Eq 1)	nm^-1^ or μm^-1^ (see text)
**SIOP**	**Specific inherent optical properties**	
$a_{\text{X}}^{*}{}_{,\text{Chla}}(\lambda )$	Chla-specific *a*_x_(λ)	m^2^ mg^-1^
$a_{\text{X}}^{*}{}_{,\text{TSM}}\left(\lambda \right)$	TSM-specific *a*_x_(λ)	m^2^ g^-1^
$a_{\text{X}}^{*}{}_{,\text{POC}}\left(\lambda \right)$	POC-specific *a*_x_(λ)	m^2^ mmol^-1^
$b_{\text{X}}^{*}{}_{,\text{Y}}\left(\lambda \right)$	Specific scattering coefficient (see $a_{\text{X}}^{*}\left(\lambda \right)$ subscripts)	see $a_{\text{X}}^{*}\left(\lambda \right)$
$b_{b\overset{*}{\text{X}},\text{Y}}\left(\lambda \right)$	Specific backscattering coefficient (see $a_{\text{X}}^{*}\left(\lambda \right)$ subscripts)	see $a_{\text{X}}^{*}\left(\lambda \right)$
**AOP**	**Apparent optical properties**	
E_u_(*z*, λ), E_d_(*z*, λ)	Up- and downwelling irradiance at depth *z*	mW m^-2^ nm^-1^
K_d_(λ or PAR)	Diffuse vertical attenuation coefficient	m^-1^
PAR	Photosynthetically active radiation (400–700 nm)	μmol photons m^-2^ s^-1^
Z_SD_	Secchi disk depth	m
R_rs_	Remote-sensing reflectance	sr^-1^

The productive season in the Baltic Sea starts with a high-biomass spring bloom of diatom and dinoflagellate algae lasting until nitrogen sources (primarily nitrate) are depleted, followed by an early summer (May-June) minimum, and subsequent summer bloom fuelled initially by phosphorus excess and increasing surface water temperature (e.g. [16]). The spring bloom is initially limited by light and progresses from south to north. The summer bloom is dominated by cyanobacteria including filamentous species with the ability to fix elemental nitrogen. Positive buoyancy of some filamentous cyanobacteria species occasionally leads to large surface accumulations, particularly during prolonged calm weather [17,18]. Phytoplankton biomass distributions generally follow nutrient availability, highest in the Gulf of Finland with an annual average up to 5 mg Chla m^-3^. Phytoplankton biomass has increased in the last decades in all sea areas except south-western basins [19].

Despite its coastal surroundings, the particle population of the Baltic Sea has a small mineral proportion compared to other coastal seas [20,21]. Suspended matter of terrestrial origin settles close to the coast [22]. Low mineral particle load combined with high CDOM concentrations results in a combination of weak light scattering and strong absorption. Consequentially, the challenges for passive optical remote sensing of this sea include low water-leaving radiance signals as well as a relatively unique combination of optically active substances.

Interpretation of water-leaving radiance for biogeochemical monitoring is ambiguous when the optical properties of the observed layer differ from those of the upper mixed layer. Due to high efficiency of light attenuation in the Baltic Sea, we may expect that the observed water layer is markedly shallower than, but under mixed conditions still representative of, the mixed layer. Under calm wind conditions, motile or buoyant phytoplankton may overcome vertical mixing and form relatively dense layers, including cyanobacterial surface accumulations for which the Baltic Sea is notorious [18]. Optical modelling previously demonstrated that the vertical distribution of phytoplankton biomass in the upper water layer does indeed significantly impact water reflectance [23]. During thermally stratified periods (in summer), detrital matter may additionally linger in the observed water layer. Lacking in situ observations representative of various degrees of vertical mixing, it is unknown to what extent remote sensing and (optical) models can accurately represent these conditions. Studies of the optical properties of the system should therefore extend to both inherent and apparent optical properties (IOPs, AOPs), and inspect whether phytoplankton is distributed homogeneously above and below the limit of remote observations.

Given the poor environmental status of the Baltic Sea ecosystem, efforts into operational optical monitoring and analysis of past and future satellite data are needed to support its cost-effective management. To this end, remote sensing algorithms that are optimally suited to the unique optical conditions of the Baltic Sea are needed. In a broader perspective, tackling the challenges in remote sensing of the dark, yet productive Baltic Sea waters will support increasingly robust algorithm development for optically extreme waters elsewhere. The new generation of operational remote sensors in the Copernicus Sentinel mission will facilitate new algorithm strategies, notably with the introduction of blue (400 nm) and red (674 nm) wavebands on the Ocean Land Colour Instrument (OLCI) on the Sentinel-3 mission, which should prove useful to characterize and quantify CDOM and phytoplankton Chla, respectively. A better understanding of the spatiotemporal variability of optically active substances is the first step in this direction and the primary focus of the present work. Because detailed optical in situ measurements from the open sea are scarce, a reference database of optical properties and associated water colour is needed for algorithm testing and development. Detailed and seasonally specific (where required) models, distributions of the underlying biogeochemical parameters, and a set of simulated remote-sensing reflectance (R_rs_) are therefore provided with this paper.

## Methods

### Sampling strategy

Optical and biogeochemical observations were compiled from eight research cruises in spring and summer in the period 2008–2012. 145 station visits were made with RV *Aranda* resulting in 459 surface water (0–17 m) samples taken with Niskin bottles on a sampling rosette equipped with a calibrated conductivity, temperature, and depth (CTD) instrument. When wave conditions allowed, samples were taken from just under the surface (0.5 m), subsurface (3 m), and depths corresponding to the top (nominally 10 m) and bottom (nominally 15 m) of any thermocline present. Under rough weather the surface samples could not always be collected. When streaks of buoyant phytoplankton (cyanobacteria) were present the research vessel was let to drift to minimize disturbance of shallow stratification. Surface accumulations were occasionally encountered in summer but always took the form of easily disturbed films rather than thick layers. Wind and wave conditions or busy ship traffic frequently necessitated the use of thrusters to stay the vessel, particularly during deep profiling. Water samples and measurements obtained close to the surface may therefore underrepresent the extent of vertical stratification of the water column.

In addition to depth profiling stations, 72 water samples were obtained while cruising from a dedicated flow-through system on board the RV with a water intake at 3 m depth. Additionally, 75 water samples collected with an automated refrigerated (4°C) water sampler on ship-of-opportunity MS *Finnmaid* of the Alg@line network were included from 17 transects between Travemünde and Helsinki, using up to 12 water samples per transect. The latter samples were primarily used to characterize the absorption by CDOM.

**Fig 1:**
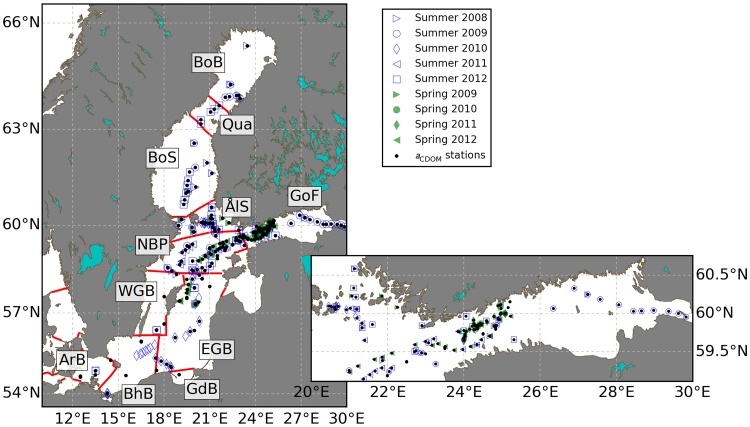
Sampling locations grouped by spring and summer campaigns. Inset: detail of locations in the Gulf of Finland. Sea areas (red lines) are divided according to HELCOM definitions, abbreviated as follows (from north to south): BoB—Bothnian Bay, Qua—The Quark, BoS—Bothnian Sea, ÅlS— Åland and Archipelago Sea, GoF—Gulf of Finland, NBP—Northern Baltic Proper, WGB—Western Gotland Basin, EGB—Eastern Gotland Basin, BhB—Bornholm Basin, ArB—Arkona Basin. Further details on the number of visits per sea area are given in Table 2.

A map of sampling locations is given in Fig 1. Samples from less-frequently visited were aggregated with adjacent areas as indicated in Table 2. The Northern Baltic Proper and the western half of the Gulf of Finland are best represented in the data set, with visits in every field campaign. For the southern Baltic Proper and the Gulf of Bothnia (Bothnian Sea, Quark, and Bothnian Bay) all samples were taken in summer with the exception of some transects of MS *Finnmaid*. Russian waters in the Eastern Gulf of Finland were only visited in August 2009. No data were collected in the Gulf of Riga and the Danish straits and only two stations are located in the Western Gotland Basin sampled in August of 2008 and 2010. Throughout this paper when referring to sample sets as ‘spring’ these were collected during cruises in April, ‘summer’ samples included July as well as August, unless specifically marked as mid-summer (July) or late summer (August). Samples taken in other months were only used to characterize CDOM.

**Table 2:** Number of site visits per season and sea area.

	Acronym	Spring bloom	Late Spring	Summer minimum	Summer bloom	Late summer	Autumn	Winter	Whole ice-free period
**(Aggregated) sea area**		**Apr**	**May**	**Jun**	**Jul**	**Aug**	**Sep-Oct**	**Nov-Dec**	**Apr-Dec**
**All areas**		64	9	25	87	60	17	29	292
Åland & Archipelago Sea	ÅlS	5	5	5	5	5	5	5	35
Gotland Basin	Got	7		4	15	5	3	5	39
Eastern Gotland Basin	EGB	7		3	14	3	2	3	32
Western Gotland Basin	WGB				1	2	1	2	6
Gdansk Basin	GdB			1					1
Gulf of Bothnia	GoB	1			11	17			29
Bothnian Bay	BoB					9			9
Bothnian Sea	BoS	1			6	8			15
The Quark	Qua				5				5
Gulf of Finland	GoF	41	4	11	19	20	4	8	107
Northern Baltic Proper	NBP	10		3	26(+1)	12	2	5	58
Southern Baltic	Sba			2	11	1	3	6	23
Arkona Basin	ArB			1	1	1	2	4	9
Bornholm Basin	BhB			1	10		1	2	14

Sampling permits were not required for operations of the Finnish research vessel in the territorial waters of Finland. In all other territorial seas and exclusive economic zones sampling permits were obtained prior to each research cruise from the respective national authorities: the Swedish coast guard, the Estonian Ministry of Foreign Affairs, the Federal Agency for Science and Innovations and/or the Ministry of Foreign Affairs of Russia, the Polish Ministry of Foreign Affairs, and the Federal Maritime and Hydrographic Agency of Germany.

### In-water measurements

Multiple depth profiles were taken at each station with a set of active optical sensors including a Wetlabs AC-S spectral absorption and beam attenuation meter with 0.1-m path length, a Wetlabs volume scattering meter (VSF, 470, 532, 660 nm each at angles 100°, 125°, 150°) and a Wetlabs BB3 fixed-angle backscattering meter (412, 595, 715 nm at 117°). A second BB3 (470, 532, 715 nm) was used in some cruises, in which case the results of overlapping wavebands were averaged. The deployment depth of each sensor was determined from its vertical distance to a pressure sensor on an attached CTD instrument (RBR XR-620 or Sea-Bird SBE 37-SI MicroCAT). AC-S measurements were processed to yield spectral absorption, beam attenuation, and scattering coefficients (respectively *a*(λ), *c*(λ), and *b*(λ) as *c*(λ)-*a*(λ)) by subtracting measurement blanks of highly purified water (measured before and after every deployment), and applying temperature and salinity corrections for the absorption and scattering of seawater. AC-S profiles were additionally subjected to visual inspection, removing measurements affected by bubbles or sensor movement causing timing errors of the internal filter wheel. Several depth profiles in early spring had to be removed due to condensation on internal optics, caused by very low (< 0°C) water temperatures. VSF and BB3 measurements were corrected for absorption (‘sigma correction’ following the manufacturer’s recommendations) and subsequently converted from fixed-angle scattering measurements to *b*_*b*_(λ) at six wavelengths (sensors combined) following integration of a polynomial function over the angle-specific measurements of the VSF, and further (and for the BB3) following [24]. Depth profiles were collected over at least the first 15 m and up to 45 m depth, simultaneously with water sampling from the sampling rosette whenever weather conditions allowed this, or otherwise within 0.5 h. Absorption measured from AC-S was used to correct other in-water measurements, whereas AC-S scattering measurements were integrated over depth bins of 1 m for comparison against absorption measured from discrete water samples (described below).

At daylight stations, spectral up- and downwelling irradiance E_u_(z,λ) and E_d_(z,λ) were recorded with TriOS Ramses-ACC-VIS sensors (320–950 nm) as a function of depth, using a reference sensor on deck to account for fluctuations in the solar irradiance arriving at the water surface. Irradiance measurements were taken from 0–15 m depth with the ship positioned so that sensors were exposed to direct sunlight. Sampling depth was measured with a pressure sensor (SBE-50, Sea Bird Electronics) attached to the sensor cage. A self-shading correction using *a*(λ) obtained with the AC-S was applied to measurements of E_u_(*z*, λ). The downwelling diffuse attenuation coefficient K_d_(λ) was calculated as the exponential slope fitted through depth profiles of E_d_(z,λ). Irradiance measurements taken over 0–7 m were used to determine attenuation of the photon flux density of photosynthetically active radiation (PAR) by first determining PAR at depth and subsequently fitting K_d_(PAR). Extrapolation of E_u_(z,λ) and E_d_(z,λ) profiles to zero-depth allowed calculation of the dimensionless subsurface irradiance reflectance.

Secchi disk depth (Z_SD_, m) was recorded with 0.5-m precision at every sampling station by lowering the 30-cm white disk from the research deck (approximately 4 m above the water line), avoiding sun glint and ship shadows.

### Laboratory analyses

Water samples were processed within two hours from sampling and kept at light intensity and temperature similar to their sampling origin until further laboratory analysis. Particulate material was concentrated onto duplicate Whatman GF/F glass fibre filters with a nominal pore size of 0.7 μm. Filters for gravimetric analysis of the dry weight of total suspended matter (TSM) were first rinsed with ultrapure water, combusted four hours at 450°C, and individually weighed prior to each cruise. Salt crystals were removed from the filters by filtering at least 50 ml of ultrapure water through the filter following filtration of sea water and before removing the filters from the filtration manifold and storing them dry in vented petri dishes. Filters for particulate organic C, N and P were acid-rinsed before washing and combustion. Sample filtrates were collected using disposable membrane filter cartridges (pore size 0.2 μm) and polycarbonate syringes without silicone stoppers.

Absorption of particulate matter was determined using the quantitative filter pad technique [25,26] using Whatman GF/F glass fibre filters (0.7 μm nominal path length). The filters were placed at the centre of the 150-mm integrating sphere accessory of a PerkinElmer Lambda800 spectrophotometer for absorbance scans at 1-nm intervals, 2-nm slit width, between 300 and 800 nm (350–800 nm for the 2008 summer cruise). Scans were converted to the absorption coefficient of particulate matter (*a*_p_(λ), units m^-1^) following a previous characterization of path length amplification for glass fibre filters at the centre of this integrating sphere, using Baltic Sea samples [27]. Further separation of *a*_p_(λ) was achieved by bleaching the filters, isolating phytoplankton pigment absorption (*a*_ph_(λ)). The bleaching procedure consisted of an 8-min exposure to a 0.1% solution of sodium hypochlorite [28,29], if necessary followed by a 70°C treatment of 80% ethanol for 5 min. The remaining fraction commonly referred to as ‘non-algal particulates’ (*a*_nap_(λ)) includes living and detrital algae and cyanobacteria, as well as other biotic particles and mineral particulate matter.

The *a*_CDOM_(λ) spectrum was measured by passing samples through 0.2-μm membrane filters and subsequent spectrophotometric determination in quartz cuvettes, against a reference of ultrapure water. Spectra were recorded over the 190–800 nm range and converted to units of absorption (log-10 to natural log conversion and normalization to 1-m path length). Absorbance results from 1 cm and longer (4, 5, or 10 cm) cells were combined to yield the best signal / noise relationship in both the ultraviolet (UV, high CDOM absorption: shorter cells) and visible to near infra-red (NIR) range.

Chla was extracted in 10 mL 96% ethanol for 24 h in darkness at room temperature [30,31]. Chla concentration was quantified on a Cary Varian Eclipse spectrofluorometer calibrated with pure Chla (Sigma) and excitation and emission slits of 5 nm centred at 430 and 670 nm, respectively. Filters for particulate organic Carbon (POC), nitrogen (PON), and phosphorus (POP) were allowed to dry and stored at room temperature (20°C) until analysis. POC and PON were measured from the same filter with a mass spectrometer (Europa Scientific). POP was determined according to [32]. Filtrates for DOC were measured by high-temperature catalytic oxidation and non-dispersive infra-red detection using a total organic Carbon analyzer (Shimadzu TOC-VCPH) equipped with chemiluminescence detector (Shimadzu TNM-1). Filters for TSM dry weight were dried overnight at 60°C and subsequently weighed before and after combustion of organic material (4 hours at 450°C) to yield total and the inorganic fraction of dry weight.

In addition to sampling stations, underway samples were taken from a dedicated flow-through system sampling at approximately 3 m depth. Chla was always quantified from these samples, whereas CDOM absorption, HPLC pigments, particulate dry weight, and organic fractions of dissolved C and N and particulate C, N and P were included depending on the specific purpose of each cruise.

Phytoplankton >2 μm were counted from 29 water samples taken during two spring cruises in 2011–2012 and 70 water samples taken during summer cruises in 2010–2012. Samples were taken from Niskin bottles at 3 m depth, fixed in acidic Lugol’s medium, stored in the dark at 4°C and left to settle in an Utermöhl chamber before examination by inverted light-microscopy. Cell numbers were converted to biomass using conversion factors of the HELCOM Phytoplankton Expert Group [33].

## Results

### Seasonality in phytoplankton community and biogeochemical composition

**Fig 2:**
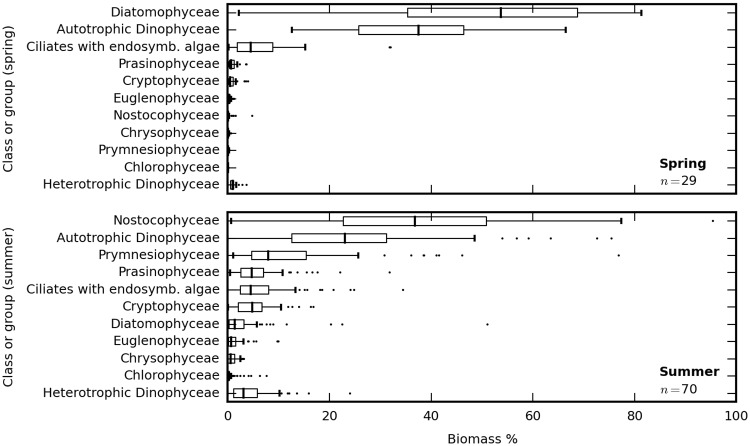
Phytoplankton community composition. Relative biomass distribution between major phytoplankton classes and the ciliate *Mesodinium rubrum* at 29 spring (top panel) and 70 summer (bottom panel) stations sampled at 3 m depth.

The dominating phytoplankton groups during spring were diatoms and dinoflagellates, accounting for >90% of phytoplankton biomass (Fig 2). Key species, defined as having maximum biomass in a single sample ≥ 5% or mean biomass ≥ 0.5% in all samples, included the diatoms *Achnanthes taeniata* (max 35.1%, mean 7.4%), *Thalassiosira baltica* (26.3%, 4.0%), *Melosira arctica* (15.9%, 2.5%), *Achnanthes* spp. (11.2%, 3.2%), *Thalassiosira levanderi* (5.4%, 1.6%), *Chaetoceros* spp. (8.3%, 1.4%), *Skeletonema marinoi* (5%, 0.6%), the dinoflagellate *Scrippsiella/Biecheleria/Gymnodinium* complex (not separated by conventional microscopy; 31.0%, 7.4%) and the dinoflagellate *Peridiniella catenata* (11.1%, 3.2%). Cyanobacteria identified as *Aphanizomenon* spp. were present in 14 out of 29 spring samples, normally with low biomass contributions (< 1%) but with exceptions in the range 1.0–4.7%. *Mesodinium rubrum*, an autotrophic ciliate with endosymbiotic algae, accounted for maximum and mean biomass contributions of 12.3% and 1.5%, respectively.

Samples taken in summer showed a more even distribution between phytoplankton classes, compared to spring (Fig 2). The most common cyanobacteria included *Aphanizomenon* spp., *Nodularia spumigena*, *Anabaena* spp. and *Ananbaena inaequalis* with mean biomass contributions of 17.7%, 5.9%, 1.4%, and 1.5%, respectively, and maximum biomass contributions up to 65.2%, 37.8%, 19.1%, and 10.6%. Among other cyanobacteria species *Snowella* spp. and *Aphanothece* spp. (occasionally identified as *A*. *paralleliformis*) were commonly observed but rarely exceeded 1% community biomass. Picocyanobacteria (predominantly *Synechococcus* sp., see [34]) were not quantified by light microscopy but may account for up to 80% of cyanobacterial biomass [35]. The most important summer dinoflagellate species were distinct from their spring community counterpart with *Dinophysis norvegica* (maximum 25.6%, mean 4.1% biomass share), *Heterocapsa triquetra* (21.7%, 2.6%), Dinophysis rotundata (4.4%, 2.3%), *Gymnodinium* spp. (3.9%, 2.3%), and *Dinophysis acuminata* (13.1, 2.3%) most commonly encountered. Diatoms generally had a minor biomass contribution in summer, mostly from the genus *Chaetoceros (C*. *wighamii*, *C*. *danicus*, *C*. *throndsenii*, *C*. *subtilis*, and *C*. *tenuissimus*). Minor contributions from unidentified centric and pennate were found in most samples at biomass contributions < 1%. *Diatoma tenuis* was abundant (13–15% biomass) at two Gulf of Bothnia stations visited in mid-July. A number of species already encountered in a large number of spring samples became abundant in summer, these included Prymnesiophyte *Chrysochromulina* spp. (max 31.6%, mean 4.8%), Prasinophyte *Pyramimonas* spp. (16.6%, 3.0%), and Euglenophyte *Eutreptiella* spp. (9.8%, 1.4%). The cryptophyte *Plagioselmis prolonga* (7.2%, 2.5%) was only abundant in summer, while the ciliate *Mesodinium rubrum* (15.7, 2.4%) still accounted for a significant share of phytoplankton biomass.

**Fig 3:**
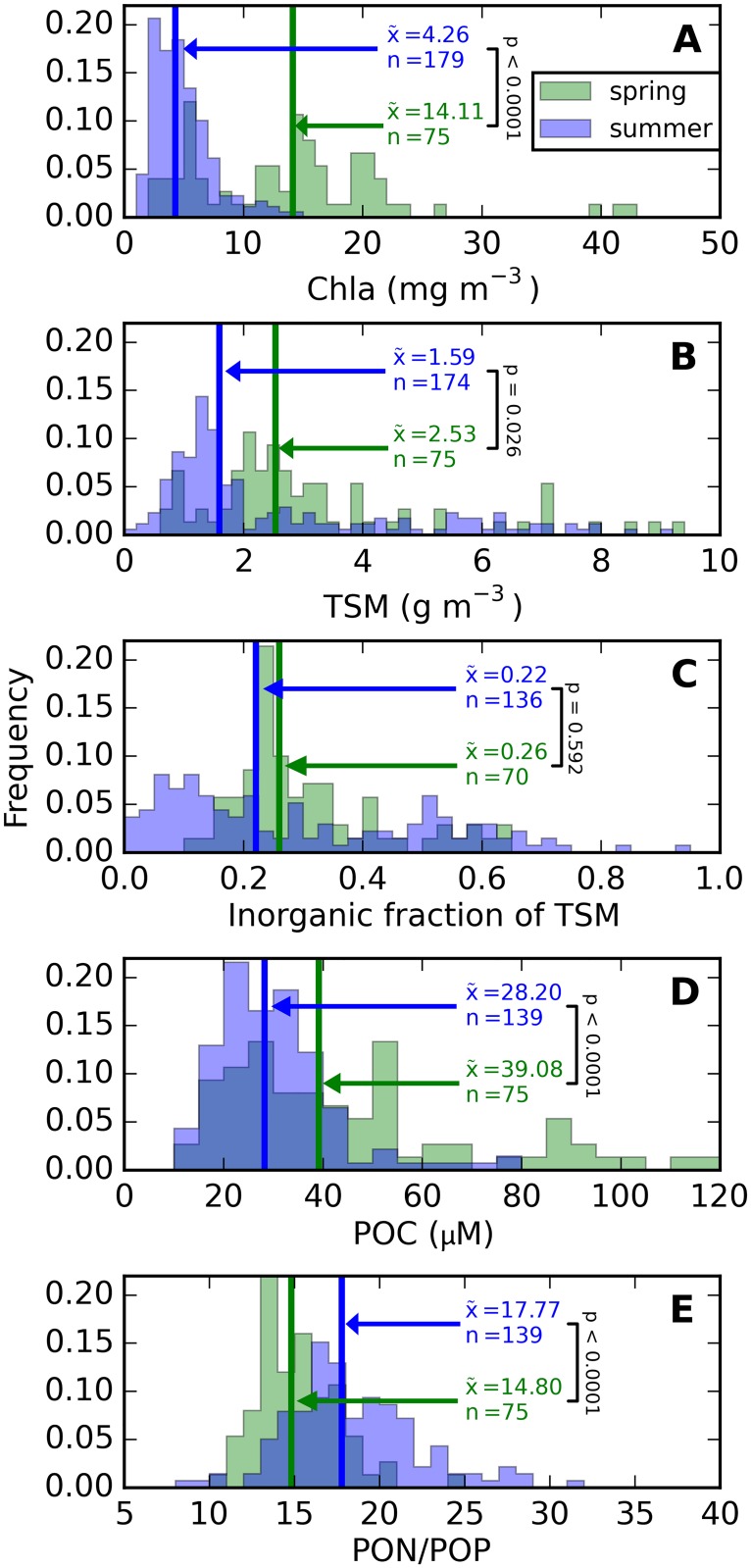
Histograms of spring and summer biogeochemical parameters. (A) Chla, (B) TSM, (C) the inorganic fraction of TSM, (D) POC, and (E) PON/POP ratio. Median values ($\bar{x}$) and sample numbers (*n*) shown with each histogram. Significant differences between spring and summer distributions are indicated by *p-*values <0.01, resulting from t-tests assuming unequal variances. Detailed data distributions are provided in the supplementary S1 Table.

Phytoplankton biomass in spring significantly exceeded summer biomass when expressed in terms of Chla (Student’s t*-*test assuming unequal variance, *p* < 0.001), TSM (*p* = 0.026), or POC (*p* < 0.001). Shapiro-Wilk tests on log-transformed data confirmed normal distributions (p > 0.01) for summer Chla, spring TSM and ITSM/TSM, spring and summer POC, and summer and all-season PON:POP ratios. Histograms of these properties allow further comparison of seasonal differences (Fig 3). Basic statistics (range, mean, median, quartiles) on these distributions are provided in the supplementary S1 Table. Spring particle communities were associated with sharply peaked ISM/TSM (Fig 3C) and significantly lower median PON:POP ratios (p < 0.001, Fig 3E) compared to broader ranges in summer, indicative of spring excess and summer depletion of bioavailable phosphorus.

### Biogeochemical relationships

**Fig 4:**
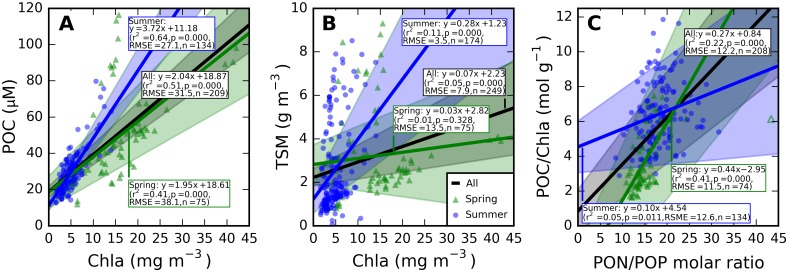
Seasonal biogeochemical relationships. (A) POC and (B) TSM to Chla, and (C) the POC:Chla ratio as a function of the PON:POP ratio. Only equations for statistically significant (p<0.01) linear least-square regressions are shown (more detail in S2 Table). One outlier for the PON:POP ratio (open circle) was excluded from the regression model.

Autochthonous production of organic matter dominated the particle population during productive periods in the open waters of the eutrophic Baltic Sea, where turbidity caused by mineral particles was already low (Fig 3B and 3C). Significant relationships can therefore be observed between POC and Chla in both spring (*r*^2^ = 0.41, *p* < 0.001, *n* = 75) and summer (*r*^2^ = 0.64, *p* < 0.001, *n* = 134) as well as in samples from both seasons combined (*r*^2^ = 0.51, *p* < 0.001, *n* = 209). The average POC:Chla ratio in summer was nearly twice as higher as in spring (Fig 4A). Relationships between TSM and Chla were weak in comparison (Fig 4B), significant but weak in summer samples (*r*^2^ = 0.11, p < 0.001, *n* = 174) but lacking a meaningful correlation in spring. Detailed analysis of differences in the POC:Chla ratio observed between the seasons (Fig 4A) revealed a significant correlation with the molar PON:POP ratio, with a relatively continuous distribution between the seasons (Fig 4C), compared to the seasonally clustered relationships between Chla and either POC or TSM.

### Diffuse attenuation relationships with reflectance and Secchi depth

**Fig 5:**
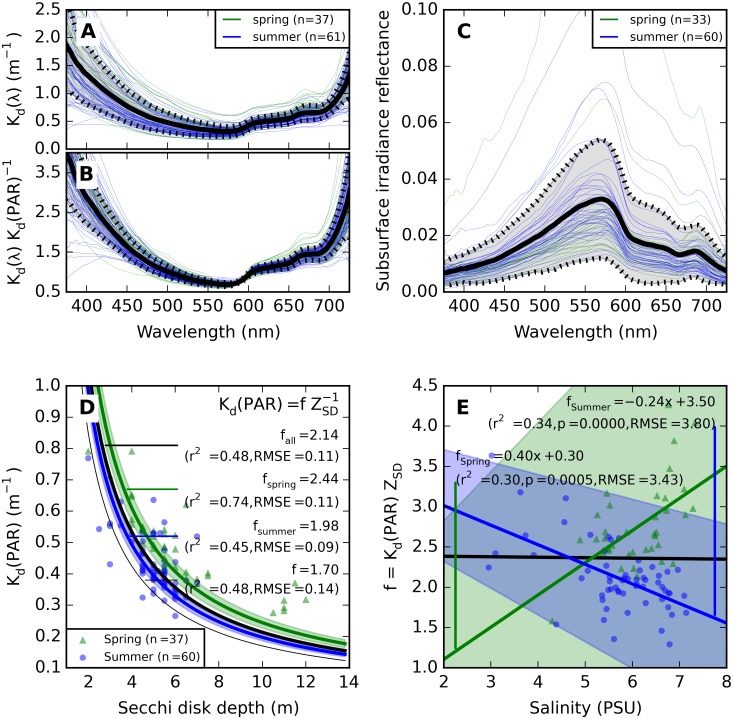
Observations and regression models of vertical attenuation and reflectance. (A) K_d_(λ), (B) K_d_ normalized to K_d_(PAR), and (C) subsurface irradiance reflectance. (D) Fits of the reciprocal model K_d_(PAR) = f / Z_SD_ to spring, summer, and combined data sets. 95% confidence intervals for the best fits of f are indicated by shaded areas. The model with coefficient f = 1.70 [36] is plotted as a thin black line. (E) Reciprocal model coefficient f as a function of salinity. Confidence intervals (95%) for the linear fits are plotted as shaded areas. Mean and standard deviation spectra (thick and dotted lines) for K_d_(λ) and K_d_(PAR) are included in the supplementary S3 Table.

In the visible spectrum, variability in K_d_(λ) was lowest between 600–700 nm (20%) and highest between 400–500 nm (38%). Phytoplankton pigment visibly influenced K_d_(675) in spring samples with high phytoplankton biomass (Fig 5A), whereas increase in K_d_(λ) at 600 nm and 700 nm corresponds to a rise in *a*_w_(λ). Despite considerable variability in the amplitude of K_d_(λ) its spectral shape was highly conserved between samples, irrespective of season. K_d_(λ) normalized to K_d_(PAR) had 10% variability at 600–700 nm and 17% at 400–500 nm (Fig 5B). The mean K_d_(λ):K_d_(PAR) ratio demonstrates that K_d_(501) = K_d_(PAR) within 1%, whereas K_d_(490), the waveband at which K_d_ is most commonly reported in remote sensing applications, exceeded K_d_(PAR) on average by 10%.

Subsurface irradiance reflectance spectra are plotted in Fig 5C. The highly conserved shape of K_d_(λ), with the absorption by CDOM and water consistently contributing to high attenuation in the short and long wavelength ends of the spectrum, resulted in reflectance spectra which were consistently low in these areas, with peak reflectance in the green spectral domain.

Models to predict K_d_(PAR) from more commonly observed Secchi disk depth were previously evaluated to hold the form K_d_(PAR) = f / Z_SD_ with f empirically found close to 1.7 ([36] and references therein). In the current data set Z_SD_ varied 2–12 m (*n* = 38) in spring and 2–7.5 m (*n* = 101) in summer. Fitting the model with f = 1.7 to the data presented here resulted in r^2^ = 0.48 and RMSE = 0.14 m, which is close to the optimal fit obtained with f = 2.14 (r^2^ = 0.48 and RMSE = 0.11 m, plotted in Fig 5C). When only spring samples were considered f = 2.44 improved the correlation but not the average error (r^2^ = 0.74, RMSE = 0.11) while for summer samples there was no marked difference using the best fit of f = 1.98 against the previously published model with f = 1.7 (r^2^ = 0.45, RMSE = 0.09). It is noted that these errors are small with respect to the precision of 0.5 m with which Z_SD_ was observed at sea. Alternative models (e.g. slope and intercept, or exponential fitting) were able to improve the fit towards clearer waters but without improved error properties over the whole range (not shown). Salinity is a poor proxy for model coefficient f (Fig 5D): a weak negative correlation with large associated error was observed between f and salinity in summer (r^2^ = 0.34, RMSE = 3.8), while the trend was opposite for spring (r^2^ = 0.30, RMSE = 3.4).

### Vertical distributions of Chla and POC and remote sensing perception

**Fig 6:**
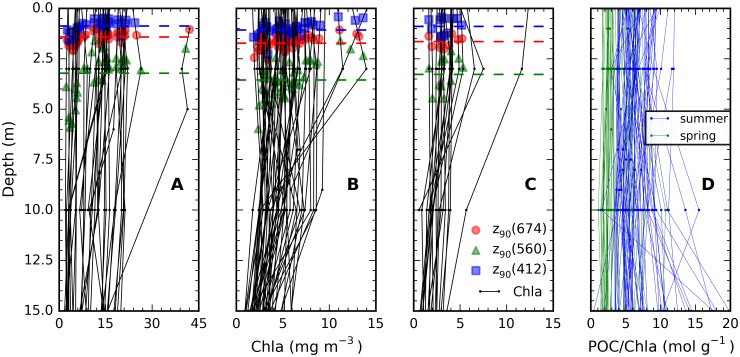
Depth profiles of Chla and the POC:Chla ratio. Chla (drawn lines) in (A) spring (April), (B) mid-summer (July), and (C) late summer (August), and (D) the POC:Chla ratio of each profile. Note different scale of Chla between panels A and B-C. The first optical depths (z_90_) at 412, 560, and 674 nm (determined as 1/K_d_(λ)) are overlaid with coloured markers; horizontal dashed lines indicate mean z_90_ for the respective period and waveband.

Vertical profiles of Chl*a* analysed from bottle samples (Fig 6) illustrate the degree of vertical inhomogeneity encountered in the water column. It is noted that, despite precautions taken to minimally manoeuvre the ship during sampling, ship-induced mixing may at times dampen vertical biomass profiles. Inhomogeneous biomass distribution was observed at some spring stations (Fig 6A) and more frequently in mid and late summer (Fig 6B and 6C). The ratio POC:Chla was relatively constant over the first 10 m depth (Fig 6D) which is consistent with results shown in Fig 4A.

Light penetration depth is overlaid in Fig 6A–6C to illustrate how representative a remotely sensed signal is of column biomass. The first optical depth (z_90_) is the inverse of the vertical diffuse attenuation coefficient K_d_, *i*.*e*. the layer depth from which 90% of the water-leaving radiance observed by a remote sensor originates [37]. The Baltic Sea attenuates blue (shown at 412 nm) and red (674 nm) light (Fig 5A and 5B) with the highest efficiency. Green light (shown at 560 nm) penetrates deepest and has the strongest dependence on phytoplankton biomass. Mean *z*_90_ values were, irrespective of season, in the order of 1.0 m (blue), 1.5 m (red), and 3.0–3.5 m (green) and only exceeded 5 m in the green band when biomass was < 5 mg Chla m^-3^. Non-parametric testing of variance in *z*_90_ between spring, mid- and late summer only showed significant differences (irrespective of wavelength) between spring and mid-summer (Kruskal-Wallis test, *F* = 1.007, *p* = 0.32). If photosynthesis is considered feasible up to the 1% light penetration depth (z_eu_ = 4.6 × z_90_), this yields a representative maximum light penetration for photosynthesis in the order of 13.8–16.1 m.

Assuming the signal above z_90_ to be representative of the full euphotic layer leads to underestimates of photosynthetic activity when biomass deeper than z_90_ exceeds biomass above this depth, and reciprocally (and more commonly) peak biomass above z_90_ may cause overestimation of column productivity if remotely sensed biomass estimates are used while the extent of mixing is not known. Table 3 lists the occurrence of peak biomass in the top (< 3 m), intermediate (3–10 m) and deepest (> 10 m) part of the surface water up to 15 m depth. In 44% of spring stations Chla biomass was equally (< 10% variation) distributed in the water column, compared to 16–25% of stations visited in mid and late summer. In 39% of spring stations and 25% mid- and 35% late-summer stations a peak in Chla biomass was found near the surface. In terms of POC, deeper (> 10 m) peaks of organic matter were found more frequently, particularly in mid-summer (18%).

**Table 3:** Depth occurrence of peak Chl*a* and POC concentrations, indicated by the number of observations (relative frequency given in brackets) where a peak in biomass distribution was evident within the observed depth interval (normally 0–15 m). Depth profiles with a coefficient of variation (CV) < 10% were considered to have no peak in vertical biomass distribution.

		Spring	Summer	
Variable	Depth of peak	April	July	August
**Chlorophyll-*a***				
< 3 m		14 (39%)	17 (35%)	5 (25%)
3–10 m		6 (17%)	16 (33%)	10 (50%)
≥ 10 m		0 (0%)	8 (16%)	0 (0%)
No peak < 15 m (CV < 10%)		16 (44%)	8 (16%)	5 (25%)
**Particulate Organic Carbon**				
< 3 m		13 (36%)	19 (48%)	10 (53%)
3–10 m		10 (28%)	10 (25%)	2 (11%)
≥ 10 m		2 (6%)	7 (18%)	0 (0%)
No peak < 15 m (CV < 10%)		11 (31%)	4 (10%)	7 (37%)

### Light absorption budgets

**Fig 7:**
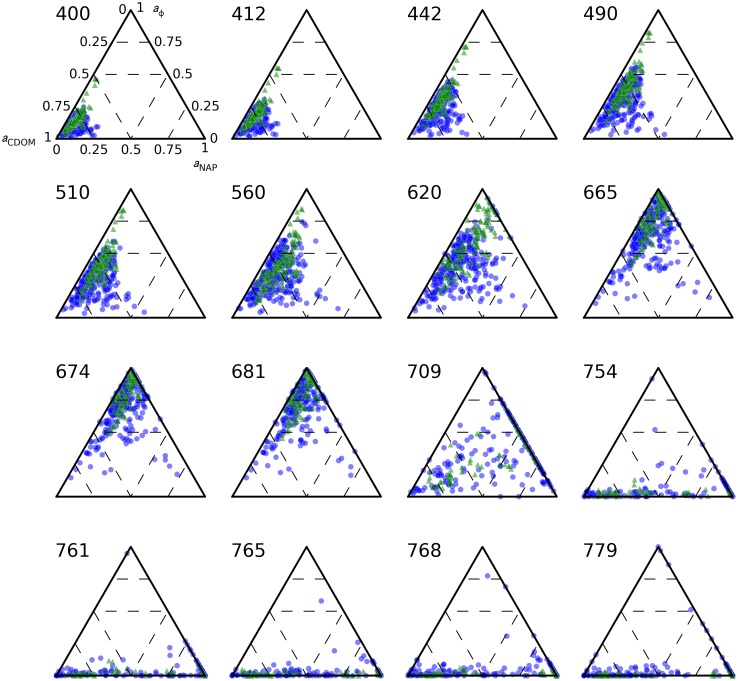
Relative contributions of CDOM, NAP, and pigments to the absorption budget. The absorption budget is shown at the central wavelength of each Sentinel-3 OLCI band (indicated to the top left of each plot). Samples with *a*(λ) < 0.01 are not plotted. Green triangles and blue circles mark observations in spring and summer, respectively.

When excluding the influence of a_w_(λ), the absorption budget of the Baltic Sea pelagic observed during spring and summer seasons was dominated by *a*_CDOM_ in blue (λ < 442 nm) and by *a*_ϕ_ at red wavebands (λ = 665–681 nm), as visualized with ternary plots for visible and NIR bands of Sentinel-3 OLCI (Fig 7). A gradual shift from *a*_CDOM_ to *a*_ϕ_ dominance occured between blue and red bands, where *a*_NAP_ occasionally contributed up to 50% in the green-yellow domain (560–620 nm). Median *a*_NAP_ ranged 5–12% (both seasons) between 400–700 nm. Median *a*_CDOM_ was 76% (±8%) at 412 nm (irrespective of season), i.e. between the > 90% reported in [5] in the Gulf of Bothnia in August (absent phytoplankton bloom), and the 38–70% range reported for the area influenced by the Oder river in autumn [6]. Median *a*_CDOM_ amounted to 81% (±7%) at 400 nm and 63% (±10%) at 442 nm. The relative contribution of *a*_ϕ_ was 84–87% in bands 665, 674, and 681 nm in spring but only 68%, 74%, and 76% respectively in these bands during summer. The difference between the seasons was not due to stronger relative contribution of *a*_NAP_ (median in the order of 7% in both seasons) but due to stronger *a*_CDOM_ contribution with a median around 16% (±16%) in summer compared to 5% (±12%) in spring samples.

### Chromophoric dissolved organic matter absorption relationships

**Fig 8:**
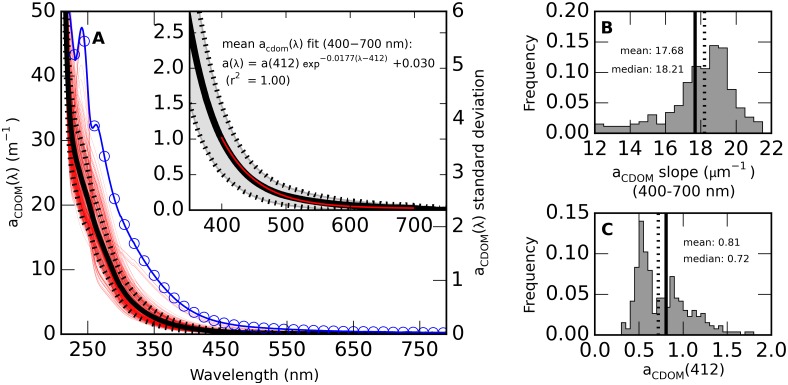
Absorption spectra and exponential model fit parameters of CDOM. (A) *a*_CDOM_(λ) spanning the UV-NIR spectrum (264 samples from 251 stations, < 10 m sampling depth). A standard deviation spectrum is superimposed (blue, circle markers). The inset of panel A shows the near-UV to visible domain as mean spectrum (thick line) ± 1 standard deviation (shaded area). The best fitting exponential model (including an offset value) for *a*_CDOM_(λ) in the 400–700 nm range is superimposed in red. Histograms of (B) slope coefficient *S* and (C) corresponding *a*_CDOM_(412) include the mean (drawn line) and median (dotted line) values. Basic statistics for data distributions, *a*_CDOM_(λ = 350–800), and spectral model coefficients are provided in the supplementary S4 Table.

The shape and magnitude of *a*_CDOM_(λ) determined from 264 samples (251 stations, April–December, Fig 8A) could be adequately described using exponential slope coefficient *S* and *a*_CDOM_ at reference wavelength λ_0_, following the commonly used exponential model (see e.g. [38]), allowing for any residual noise or scattering with intercept *k* [39]:

$$a_{\text{CDOM}}\left(\lambda \right)=\;a_{\text{CDOM}}\left(\lambda_{0}\right)e^{-S\left(\lambda -\lambda_{0}\right)}+k$$ (1)

The mean *a*_CDOM_(λ) spectrum was well captured by the model over the 400–700 nm range using λ_0_ = 412 nm (r^2^ = 1.00), with *S*(400–700) = 0.0177 nm^-1^ and *k* = 0.030. Considerable variability in the UV region reflects the variable presence of chromophores from terrestrial and autochthonous origins, with associated peaks in the standard deviation spectrum (Fig 8A). This variability is not observed in the visible domain (Fig 8A inset) and therefore not explored here. *S* is here expressed as the slope measured over the 400–700 nm range. Variability in *S* (Fig 8B) and *a*_CDOM_(412) (Fig 8C) was substantial, showing a short-tailed distribution of S (mean = 0.0177, median 0.0182 nm^-1^) and a bimodal distribution of *a*_CDOM_(412), the latter having modes at 0.53 m^-1^ (central and southern sea areas) and 0.85 m^-1^ (Gulf of Bothnia, Eastern Gulf of Finland).

**Fig 9:**
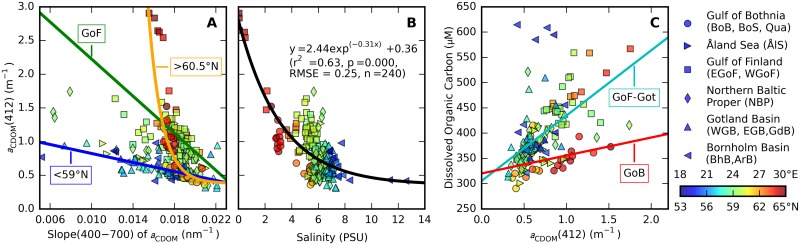
Optical-biogeochemical relationships of CDOM. Marker colour corresponds to longitude in the Gulf of Finland (square markers) and to latitude in other sea areas. Marker shape indicates sea region (see legend). (A) *a*_CDOM_(412) as a function of the exponential slope coefficient *S* (Eq 1) determined over the 400–700 nm range. Regression models plotted as thick lines are given in the text and correspond to three regions: stations south of 59°N, the Gulf of Finland, and stations north of 60.5°N. (B) Salinity as a function of *a*_CDOM_(412). An exponential regression model fit is given by the thick drawn line. (C) DOC as a function of *a*_CDOM_(412), with linear regression model fits to samples from the Gulf of Bothnia (GoB), and all other sea areas including Åland Sea / Archipelago Sea but excluding Borhnolm Basin (labelled Gof-Got). Regression models are described in the main text.

Relationships between *a*_CDOM_ magnitude and *S* are expected to have a geographical component due to variant run-off from river catchments and subsequent dilution and transformation in the lifetime of CDOM in the sea. In the Baltic Sea the CDOM riverine input is particularly strong in the eastern Gulf of Finland and the semi-isolated Gulf of Bothnia. Indeed, three models were found to represent the relation between *a*_CDOM_(412) and *S*(400–700) between these sea areas and the rest of the sea. For stations south of 59°N (Fig 9A, blue line) the linear model *a*_CDOM_(412) = -33.36 *S* + 1.16 provided an adequate fit to the data (r^2^ = 0.45, *p* < 0.001, RMSE = 0.12 m^-1^, *n* = 75). For stations in the Gulf of Bothnia, north of 60.5°N (Fig 9A, orange line) the exponential model *a*_CDOM_(412) = 3.64 105 *e*^(-766 *S*)^ + 0.38 performed reasonably well (r^2^ = 0.52, *p* < 0.001, RMSE = 0.21 m^-1^, *n* = 33), although the low number of samples and some discontinuity in *a*_CDOM_(412) warrants caution in using this model. Interestingly, samples from the eastern Gulf of Finland (in the Neva river bay) with *a*_CDOM_(412) > 2.5 m^-1^ are better described by this model than by the linear model for all samples taken in the Gulf of Finland (Fig 9A, green line), which has the shape *a*_CDOM_(412) = -137.66 *S* + 3.60. The GoF model is the weakest of the three models (r^2^ = 0.22, *p* < 0.001, RMSE = 0.40 m^-1^, n = 84) and illustrates the presence of complex mixing dynamics with multiple riverine inputs to this area [11].

Salinity and *a*_CDOM_(λ) can be used interdependently to trace water masses in coastal seas (*e*.*g*. [40]). Correlations between a_CDOM_(λ) and salinity have been established in the southern Baltic Sea [12,28] and to trace water masses in the Baltic-North Sea exchange [41,42]. In this census of optical properties of the open Baltic Sea, *a*_CDOM_(λ) and salinity are fairly well correlated (Fig 9B, r^2^ = 0.63, *p* < 0.001, RMSE = 0.25 m^-1^, *n* = 240) although a high degree of scatter is observed in the western Gulf of Finland. Some degree of seasonality, as previously found for waters of the southern Baltic Sea [12], will be evident but remains to be studied in detail, including variability in absorption properties in the UV. Exploration of correlations between *S* and salinity or between *a*_CDOM_(412), *S*, and the distance to major rivers did not yield patterns which could be exploited in statistical optical models.

The ultimate applications of a_CDOM_(λ) mapping coupled to biogeochemical ecosystem modelling are to predict underwater light availability for photosynthesis, and to trace DOC. Once *a*_CDOM_ is retrieved from a remote optical sensor at a reference (blue) waveband, the models described above for *S* and salinity will be useful to determine *a*_CDOM_(λ) with relatively high confidence. Subsequently, a model relating DOC to *a*_CDOM_ is required. Two models could be described to capture the variability in this relationship. For the combined sea areas in the Gulf of Bothnia, the linear model DOC = 35.54 *a*_CDOM_(412) + 320.25 was not highly significant (r^2^ = 0.15, *p* = 0.05, RMSE = 38.5 μM, *n* = 26), possibly due to small sample size. A model for the other sea areas excluding the Bornholm Basin follows DOC = 129.17 + 305.62 and was significant (r^2^ = 0.64, *p* < 0.001, RMSE = 131.8 μM, *n* = 114) although a high RMSE is noted. Similar intercept values between the models suggest that the endpoint of CDOM transformations (unlikely to be reached due to prevalent mixing) is in the order of 305–325 μmol L^-1^.

### Particulate absorption relationships and pigment packaging

**Fig 10:**
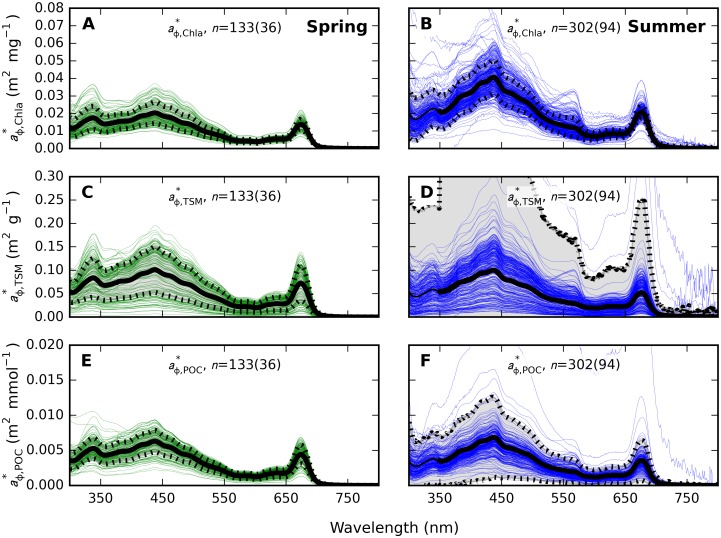
Pigment absorption spectra. Pigment absorption from samples taken at <20 m depth normalized to (A-B) Chla, (C-D) TSM, and (E-F) POC in (A, C, E) spring and (B, D, F) summer samples. The number of samples and stations (in brackets) is indicated in each plot. Thick lines mark mean spectra whereas shaded areas bounded with dashed lines give the standard deviation. The mean spectrum was forced to show no discontinuity at 350 nm, to correct for lacking data in the 300–350 nm range for samples from the summer cruise in 2008. See also supplementary S8 Table.

**Fig 11:**
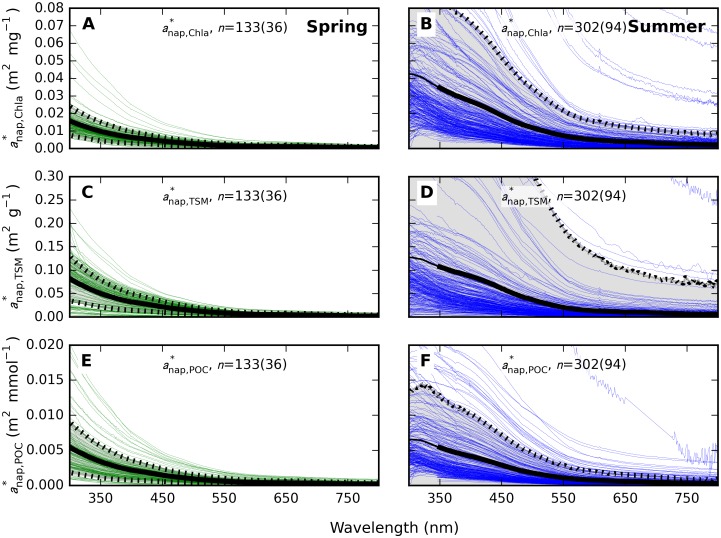
Non-algal particle absorption spectra. The NAP absorption fraction from samples taken at < 20 m depth, normalized to (A-B) Chla, (C-D) TSM, and (E-F) POC in (A, C, E) spring and (B, D, F) summer samples. Plot decorations and axis scaling are as in Fig 10. See also supplementary S9 Table.

Seasonal differences in particulate SIOPs were most prominent for absorption expressed per unit of Chla, *i*.*e*. as $a_{\phi }^{*}{{}_{,}}_{\text{Chla}}\left(\lambda \right)$ (Fig 10A and 10B) and $a_{\text{nap}}^{*}{{}_{,}}_{\text{Chla}}\left(\lambda \right)$ (Fig 11A and 11B). Mass-normalized results for *a*_p_(λ), the sum of *a*_ϕ_ and *a*_nap_, are not plotted here but supplementary S7, S8 and S9 Tables include SIOP data for all of $a_{\text{p}}^{*}\left(\lambda \right)$, $a_{\phi }^{*}\left(\lambda \right)$, and $a_{\text{nap}}^{*}\left(\lambda \right)$. Spring and summer $a_{\phi }^{*}{}_{,\text{Chla}}\left(\lambda \right)$ were significantly different (t-test, unequal variance, two-tailed *p*-value << 0.001) throughout the spectrum.

Comparing mean $a_{\phi }^{*}{}_{,\text{Chla}}(440)$ of spring and summer particle populations, we observed $a_{\phi }^{*}{}_{,\text{Chla}}(440)$ twice as high in summer (0.040 (±0.010) m^2^ mg^-1^) compared to spring (0.020 (±0.006) m^2^ mg^-1^). The difference was smaller at 675 nm with 0.014 (±0.003) m^2^ mg^-1^ in spring compared to 0.021 (±0.004) m^2^ mg^-1^ in summer. The variation in the spectral magnitude (average $a_{\phi }^{*}{}_{,\text{Chla}}$ over 400–700 nm) was similar between the seasons with a coefficient of variation (CV) of 26% in spring (*n* = 133 samples from 36 stations) compared to 28% (*n* = 302 samples, 94 stations) in summer. The presence of phycoerythrin (absorption peak at 560 nm) was noted only in summer, likely associated with *Synechococcus* sp. This pigment peak was also more distinct in samples taken at the bottom of the euphotic zone (10–20 m), although depth was otherwise a minor source of variability in absorption properties and it is not explored further here.

A wider range of variability was observed in $a_{\phi }^{*}{{}_{,}}_{\text{TSM}}\left(\lambda \right)$ (Fig 10C and 10D) with spectrally averaged CV = 50% in spring and 392% in summer. There was no statistical difference (t-test) at any wavelength between the spring and summer sets and mean $a_{\phi }^{*}{{}_{,}}_{\text{TSM}}\left(440\right)$ in spring (0.100 m^2^ g^-1^) and summer (0.099 m^2^ g^-1^) were near identical, while summer $a_{\phi }^{*}{{}_{,}}_{\text{TSM}}\left(675\right)$ was lower (0.052 m^2^ g^-1^) than in spring (0.072 m^2^ g^-1^) which contrast the results given for $a_{\phi }^{*}{{}_{,}}_{\text{Chla}}\left(675\right)$.

Normalization to POC (Fig 10E and 10F) yielded even smaller differences between mean spring and summer spectra with $a_{\phi }^{*}{}_{,\text{POC}}(440)\;=\;0.006$ m^2^ mmol^-1^ in spring and 0.007 m^2^ mmol^-1^ in summer, and $a_{\phi }^{*}{}_{,\text{POC}}(675)\;=\;0.004$ m^2^ mmol^-1^ in both spring and summer. A large degree of variability (CV = 84%) in the magnitude of $a_{\phi }^{*}{}_{,\text{POC}}$ was observed in summer (CV = 27% in spring), which may cause the difference between spring and summer $a_{\phi }^{*}{}_{,\text{POC}}(\lambda )$ sets to be statistically significant (t-test, unequal variance, two-tailed *p* < 0.001) at all wavebands except for green (550–580 nm) and NIR minima, despite the similarity in shape and magnitude of the seasonal mean spectra.

Variability in $a_{\text{nap}}^{*}$ (Fig 11) was higher in summer compared to spring, regardless of whether normalization followed the concentration of Chla (summer CV = 195%, spring 59%), TSM (summer CV = 785%, spring 57%) or POC (summer CV = 158%, spring 69%). The spectral variability of *a*_nap_(λ) was modest in both seasons. The spectral slope of *a*_nap_(λ), fitted over 400–700 nm using the same model as for *a*_CDOM_(λ) (Eq 1), varied in the range 0.0049–0.0126 nm^-1^ in spring with median 0.0097 nm^-1^ and mean (± standard deviation) of 0.0089 (± 0.0016) nm^-1^. In summer, the range spanned 0.0031–0.0144 nm^-1^ with a median value of 0.0103 nm^-1^ and mean 0.0098 (± 0.0021) nm^-1^. Summer samples did occasionally show a less uniform decline of absorption with wavelength in the blue domain, which may suggest remnant pigment absorption despite rigorous bleaching procedures. Detailed data distributions of the slope of *a*_nap_(λ) are given in the supplementary S2 Table.

**Fig 12:**
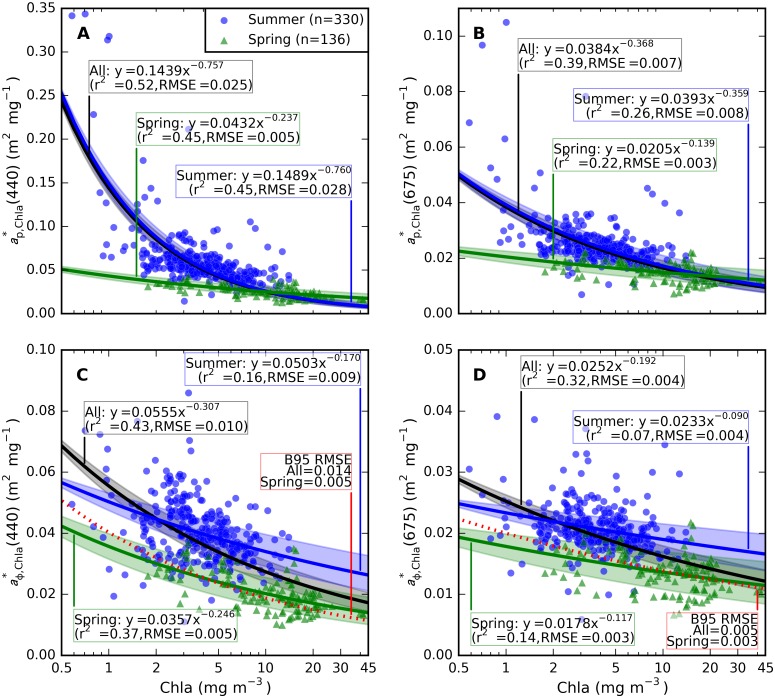
Pigment packaging effect on Chl-specific absorption coefficients. (A) $a_{\text{p}}^{*}{}_{,\text{chla}}(\text{440})$ (B) $a_{\text{p}}^{*}{}_{,\text{chla}}(\text{675})$, (C) $a_{\phi }^{*}{}_{,\text{chla}}(\text{440})$ and (D) $a_{\phi }^{*}{}_{,\text{chla}}(\text{675})$. Power model fits to spring, summer, and observations in both seasons combined plotted as blue, green, and black lines, respectively. Shaded areas in corresponding hues indicate 95% confidence limits for these models. All models were significant at *p* < 0.001. The model for $a_{\phi }^{*}{}_{,\text{chla}}(\lambda )$ of [43] is included for reference (B95) in panels C and D, along with RMSE corresponding to fits of the present data (spring and all combined) to this model. Supplementary S11 Table contains regression fits at all wavelengths.

Pigment packaging is expressed as a reduction in pigment absorption efficiency with increasing cellular pigment concentration. While the influence of cell size and morphology is not explored in this study, the wide range of pigment concentrations encountered in the data set makes it possible to express a spectral model for the effects of pigment packaging on $a_{\text{p}}^{*}{}_{,\text{chla}}(\lambda )$ and $a_{\phi }^{*}{}_{,\text{chla}}(\lambda )$. The effect is strongest when $a_{\text{p}}^{*}{}_{,\text{chla}}(\lambda )$ is considered, as shown in Fig 12A and 12B at 440 and 675 nm, respectively. Spring and summer response functions can be seen to deviate at low and intermediate Chla concentrations (0.5–5 mg m^-3^). Power function regression fits capture the response at both plotted wavebands with acceptable coefficient of determination and error properties, although summer samples dominate the model that is given for the seasons combined.

The best-fitting power function applied to spring observations of $a_{\phi }^{*}{}_{,\text{chla}}(\lambda )$ (Fig 12C and 12D) corresponds well to the frequently applied model (B95) of [43]–either model would result in a root mean square error (RMSE) of 0.005 m^2^ mg^-1^ for $a_{\phi }^{*}{}_{,\text{chla}}(\text{440})$. The error increases approximately three-fold to 0.014 m^2^ mg^-1^ when both spring and summer observations are considered against B95, compared to 0.010 m^2^ mg^-1^ for the best fitting model. The B95 model did not significantly describe the package effect in summer observations alone at 440 nm. The model errors are smaller when $a_{\phi }^{*}{}_{,\text{chla}}(\text{675})$ is considered (Fig 12D). The B95 model then has an associated error of 0.005 m^2^ mg^-1^ against all samples combined, compared to 0.004 m^2^ mg^-1^ of the best-fitting model. This error is likely acceptable, particularly considering the high amount of scatter around of the data around the models and low associated coefficients of determination (r^2^ = 0.14, 0.07, and 0.32 respectively for spring, summer, and both seasons).

### Particulate (back)scattering relationships

**Fig 13:**
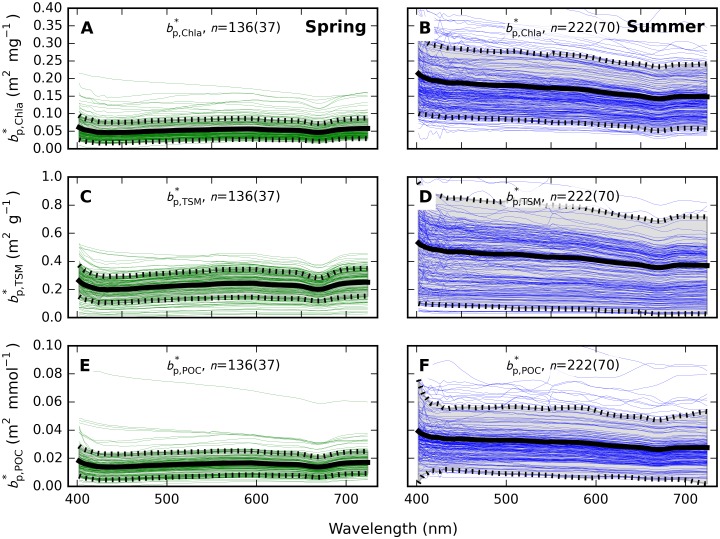
Particulate scattering spectra. Scattering normalized to (A-B) Chla, (C-D) TSM dry weight, and (E-F) POC in spring and summer, respectively. Samples from up to 4 depths < 20 m are plotted per station, corresponding to sampling depths for discrete water samples. The number of samples followed by the number of stations (in brackets) is indicated in each plot. Spectral mean and standard deviation of the data plotted here are available in supplementary S10 Table.

Particulate scattering *b*_p_(λ) in spring showed a negligible spectral slope but a distinct influence of pigment absorption, with ±10% variation around the spectral average. Spectral variability was most visible at the blue and red peaks of Chl*a* and readily observed in plots of $b_{\text{p}}^{*}(\lambda )$ (Fig 13), similar to earlier reports [44–46]. The relative spectral variation around pigment absorption peaks was minor in summer, probably owing to higher magnitude of $b_{\text{p}}^{*}(\lambda )$. A more distinct spectral slope of -0.11% nm^-1^ (between 400 and 700 nm) was, however, observed in summer samples.

**Fig 14:**
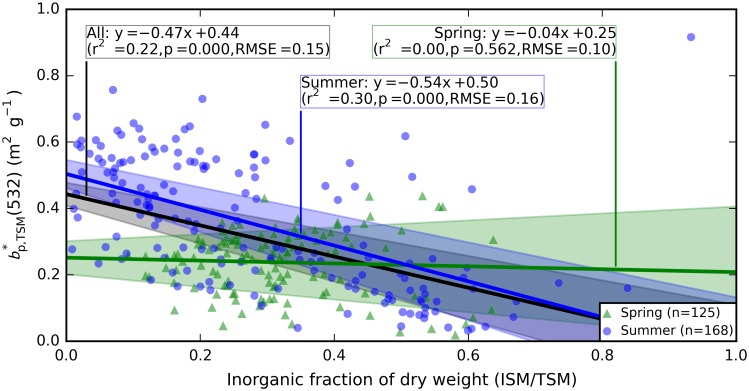
TSM-specific particulate scattering as a function of the inorganic fraction of particulate dry weight. Regression models are annotated for all samples (black line), spring samples (green line), and summer samples (blue line). Shaded areas in corresponding hues indicate the 95% confidence interval of the model. Regression model statistics are described in full in the supplementary S2 Table.

Despite clear differences in the magnitude of $b_{\text{p}}^{*}(\lambda )$ between the seasons, within-season variability was similar. In spring, the spectrally averaged CV was 56%, 42%, and 56% for normalization of *b*_p_(λ) to Chla, TSM, and POC, respectively. In summer, $b_{\text{p}}^{*}{}_{,\text{chla}}(\lambda )$ was equally variable (56%), whereas $b_{\text{p}}^{*}{}_{,\text{tsm}}(\text{86\%})$ and $b_{\text{p}}^{*}{}_{,\text{POC}}(\text{75\%})$ had wider variability. Chla concentration is thus a consistent indicator of the variability in the particulate scattering SIOP in both bloom seasons. If particles with a high inorganic content played a significant role in scattering, we would expect $b_{\text{p}}^{*}(\lambda )$ to show an increase with inorganic particulate dry weight. Instead, a weak (negative) trend of $b_{\text{p}}^{*}{}_{,\text{TSM}}(\text{532})$ with an increasing proportion of ITSM is observed, and only when summer samples are considered (Fig 14, r^2^ = 0.30, p < 0.001, RMSE = 0.16 m^2^ g^-1^, n = 168).

**Fig 15:**
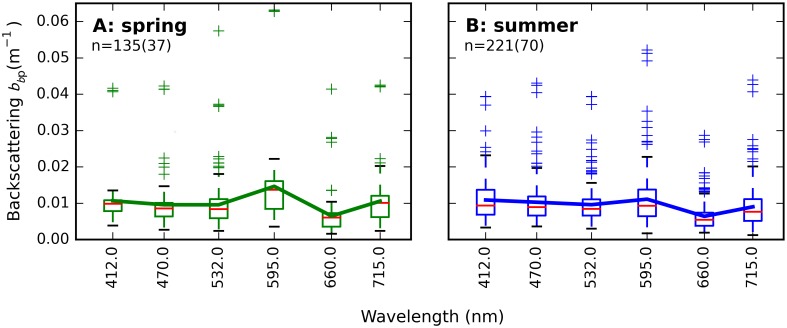
Particulate backscattering *b*_*bp*_(λ). ***b*_*bp*_** is shown for up to 6 wavebands (depending on combination of sensors deployed) and up to 4 depths (< 20 m) per station from (A) spring and (B) summer cruises. One observation in panel A with values up to 0.1 m^-1^ is not visible. The 532 nm band includes measurements at both 530 and 532 nm. Boxplots are drawn from the lower to the upper quartiles around the median (red bar), with whiskers indicating 1.5 times the quartile range, and any other values plotted as fliers. Basic statistics on *b*_*b*p_(λ) are included in supplementary S5 Table.

Spectral variation in particulate backscattering (*b*_*b*p_(λ)) is presented as boxplots (Fig 15) to accommodate for incomplete overlap in the waveband sets of VSF and BB-3 sensors deployed during various cruises. Backscattering spectra were similar in shape to $b_{\text{p}}^{*}(\lambda )$ shown in Fig 13, with influence of pigment absorption particularly visible in the red channel at 660 nm. A minor peak at 595 nm is visible from observations in spring. The observed range is similar to that reported previously for the Baltic Sea in the Gulf of Finland [47]. Spring and summer *b*_*b*p_ spectral means were not significantly different.

**Fig 16:**
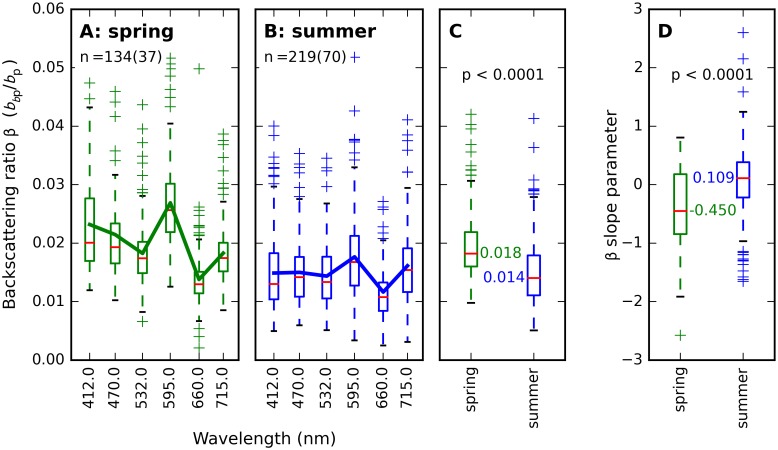
Particulate backscattering ratio *β* (b_bp_/b_p_). (A) spring (134 observations at 37 stations) and (B) summer (219 observations, 70 stations). Boxplots are displayed as quartiles around the median, drawn lines connect mean values. (C) Significant differences between the season-specific distributions of the spectrally averaged β and (D) spectral slope of β are observed. See supplementary S6 Table for the data distributions plotted here.

The particulate backscattering ratio (β_p_(λ) = *b*_*b*p_(λ)/*b*_p_(λ)) was calculated for all wavebands where *b*_*b*p_(λ) was available. Spectral variation identified in *b*_*b*p_(λ) and *b*_p_(λ) was amplified in β(λ) as shown in Fig 16A and 16B. Higher $b_{\text{p}}^{*}(\lambda )$, offset against generally lower constituent concentrations in summer (Fig 3) and seasonally similar *b*_p_(λ) resulted in net higher backscattering ratios in spring than in summer, with spring β(532) close to the commonly used values of Petzold’s volume scattering function for San Diego Harbor at the same angle and wavelength [48]. Both the spectral mean (Fig 16C) and spectral slope (Fig 16D) of β were significantly different between seasons.

**Fig 17:**
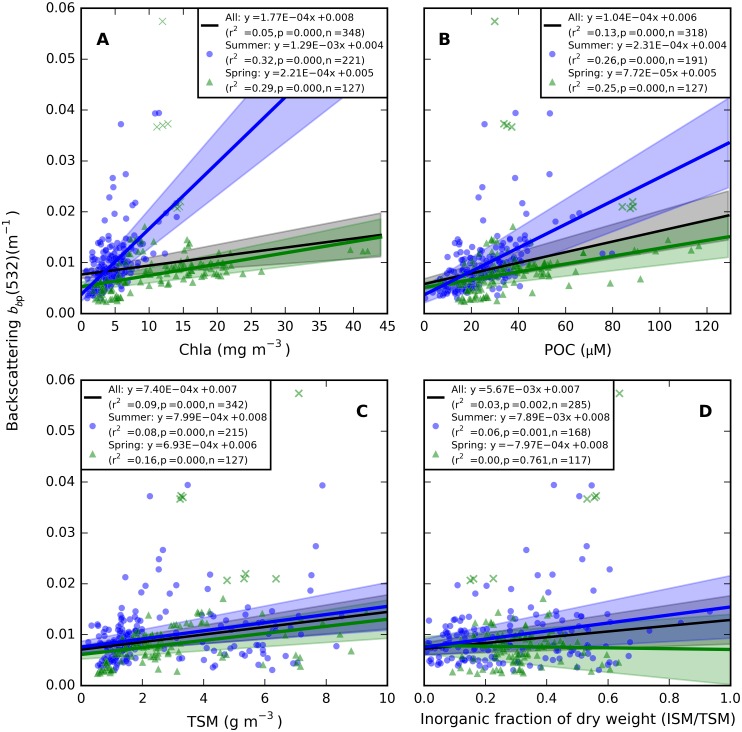
Particulate backscattering *b*_*b*p_(532). Plotted for 4 depths < 20 m per station, as a function of (A) Chla, (B) POC, (C) TSM, and (D) the proportion of ISM in TSM. Drawn and dotted lines are the linear regression fit to the data for spring (green), summer (blue) and all plotted stations, respectively. Eight observations from stations with a stronger coastal influence (marked as crosses) were excluded from the linear fit for spring.

Weak but significant season-specific correlations were found between *b*_*b*p_(532) and Chla, POC, and TSM (Fig 17). Seasonal effects were least evident with TSM and POC. The poorest correlations were found with the inorganic fraction of suspended matter (ISM/TSM), suggesting an absence of inorganic particles in most observations, while mineral matter may be contributed by silicates from diatom frustules present in the ash fraction.

## Discussion

The Baltic Sea is perhaps most widely known for its occasional magnificent displays of cyanobacterial blooms, decorating surface waters during periods of calm weather in summer, and clearly visible from space [18,49,50]. The prolonged calm weather leading to surface accumulation [17] is however not as common as occasional wind mixing, and summer blooms are therefore usually dispersed between the water surface and thermocline (Fig 6, Table 3). Optical observations presented in this study include relatively few encounters with cyanobacteria accumulated at the surface. Correspondingly, we have not observed marked optical variability over depth within the euphotic zone. Marginally wider variation with depth was observed in relation to the absorption signature of the phycoerythrin pigment in summer, likely associated with pico-cyanobacteria [51]. This, however, will have minimal impact on remote sensing studies observing only the first optical depth. The prevalence of both filamentous and pico-cyanobacteria in summer is nevertheless a prominent factor in the optical seasonality of the Baltic Sea.

Spring blooms develop significantly higher POC in the mixed layer than summer blooms (Fig 3D) and even three-fold higher Chla (Fig 3A). The difference in the Chla to POC ratio is explained by a less prominent role of Chla in photosynthetic light harvesting in cyanobacteria (which are only prevalent in summer) compared to algae [51–53]. Limited variability in spring $a_{\phi }^{*}$ and $a_{\text{nap}}^{*}$ SIOPs, regardless of whether normalization of the IOPs follows Chla, TSM, or POC concentration (Figs 10 and 11), is a strong indication that absorption is governed by the phytoplankton component. Variability in summer IOPs is better explained by POC and Chla than by TSM concentration, supporting our understanding that phytoplankton are the main source of variability in particulate absorption, while detrital matter is a more variable component in summer. We also observe that within-season correlations between Chla and TSM are well established (Fig 4B) and inorganic TSM does not act as a prominent driver of scattering SIOPs.

The Baltic Sea spring bloom builds up as light availability increases, and dissipates soon after nutrient depletion as particles sediment out. Consequently, the spring bloom has an intense but short-lived impact on the optical properties of the surface layer [54,55]. In contrast, thermal stratification in summer causes the summer particle population to linger in surface waters. We understand this to lead to a relatively complex and variable particle composition in summer compared to our observations during spring bloom. We observed a higher proportion of absorption associated with the non-algal particle fraction of particles (Fig 7), wider variability in IOPs between stations (Figs 10, 11 and 13) and a relatively diverse phytoplankton community (Fig 2) in summer compared to spring. Inorganic nutrient availability, particularly bio-available phosphorus, is typically at minimum detection levels during summer sampling (not shown), suggesting that after the initial establishment of the phytoplankton population its biomass is fuelled by recycling of nutrients. The more significant contribution of *a*_nap_(λ) to light absorption in summer compared to spring is therefore likely associated with a larger proportion of detrital material, in turn contributing to nutrient recycling and prolonged blooms. Reciprocally, our optical measurements support our understanding that particulate matter produced in spring is mixed deeper and met with faster export from the visible surface layer. It would be of interest to bio-optical modelling and remote sensing efforts to further differentiate the absorption and scattering SIOPs of phytoplankton, detritus, and mineral particulates so that the influence of seasonality in phytoplankton optical properties and the prevalence of detrital material can be investigated separately. This will likely require a multivariate statistical approach to an even larger and seasonally resolved data set than presented here.

Absorption of light by CDOM dominates the underwater light climate even during the most productive periods in the Baltic Sea and has a marked influence on the absorption by water constituents, even in satellite wavebands in the 600–665 nm range (Fig 7). Given that (1) the predominantly organic particle population observed here does not give rise to strong particulate backscatter, and (2) *a*_CDOM_ in the blue-red and *a*_w_ at >600 nm provide an efficient absorption medium and weak water-leaving radiance, reflectance is always expected to peak in the green domain between 560–600 nm. Reflectance simulated from the presented SIOP models (see supplementary S1 Appendix and S1 Dataset) can be used to confirm this behaviour over and beyond the range of observed constituent concentrations. In fact, we would only expect the typical green-peaked reflectance signature of the open Baltic Sea to change when surface accumulations of cyanobacteria significantly enhance near-infrared reflectance.

Combining our observations made thus far, we find that the pelagic zone of the Baltic Sea presents a relatively unique combination of weak particulate backscattering originating primarily from organic particulates, and efficient light absorption by CDOM and water in a large part of the UV to NIR domain. This results in weakly reflecting waters where the absorption signature of phytoplankton is easily masked by CDOM absorption. The implication for remote sensing is that algorithms designed to retrieve phytoplankton Chla biomass by isolating the pigment absorption properties may prove considerably less sensitive in quantifying the pigment than algorithms which are sensitive to light scattering by suspended matter. Because the open Baltic Sea particle population is largely associated with phytoplankton (at least in the productive season), there is a real risk of developing the latter type of algorithm when statistical performance, rather than specificity to phytoplankton pigment in the presence of non-phytoplankton scattering matter, is used as the primary performance criterion.

The dataset described here features rich seasonal diversity in optical properties but also allows us to determine where further investigations of optical properties and associated remote sensing challenges, will be useful. A few observations on where further challenges lie, are given here. In terms of seasonality, our observations focus on the peak of biomass production in spring and early summer, with some additional late summer (august) observations. These periods of peak phytoplankton abundance exhibit significant seasonal differences in IOPs and derived optical-biogeochemical models, which are governed by phytoplankton particles over a wide range of Chla and POC concentrations. It is not yet clear whether non-phytoplankton particulates, in particular those with higher scattering efficiency, play a more significant role in the absorption and scattering budget of the open Baltic Sea when we consider other periods than those of peak periods of phytoplankton growth. In other seasons, including the early summer minimum observed in May-June, we expect to observe further variability in IOPs, either increasing the observed complexity in optical properties for remote sensing algorithm development or, hopefully, leading to a better characterization of gradual optical transitions. Late-bloom succession of heterotrophic plankton is also poorly optically characterized at present, and would likely increase the relative importance of *a*_nap_(λ) outside peak phytoplankton growth periods. Similarly, autochthonous production of CDOM is associated with bloom degradation and detailed studies of seasonal autochthonous DOM production would lead to better characterization of (regional) relationships between *a*CDOM and dissolved matter composition than established here. For the benefit of developing seasonally valid optical monitoring strategies it would therefore be highly useful to include IOP measurements in regular sampling activities, even if focussed on relatively few observation stations.

The role of small versus large sized particles, flocculation, and colony formation could not be defined in this study of bulk (non-particle specific) optical properties. The particle size distribution is known to influence pigment absorption efficiency [43] and would likely help explain the variability observed in the pigment packaging effect, both within and between seasons (Fig 12). A weak dependence of $b_{\text{p}}^{*}{}_{,\text{TSM}}(532)$ and $b_{\text{bp}}^{*}{}_{,\text{TSM}}(532)$ on the inorganic fraction of TSM (Figs 14 and 17D) may be associated with aging of detrital organic matter in summer stratified surface water, where flocculation associates inorganic matter with larger particles for which we expect lower mass-specific backscattering. Total particulate scattering was higher in summer than in spring whereas backscattering was of similar intensity (Figs 15 and 16). While it is impossible to determine the particle-specific backscatter in our data set because key measurements are lacking (e.g. using flow cytometry, laser diffraction, particle size distributions to obtain individual particle properties), it is clear that summer (cyanobacteria-dominated) particle population were associated with significantly lower backscattering ratios. Cyanobacteria have limited internal structure and are therefore expected to have weak backscatter [56], although species which develop gas vacuoles would elevate backscattering efficiency [57]. There is still controversy in recent literature regarding the backscattering efficiency that should be associated with cyanobacteria blooms, and future remote sensing studies should ideally take into account the morphology of the dominant species before assigning scattering IOPs from the limited body of literature available on this topic.

In conclusion, optical and biogeochemical observations made over a five-year period in productive seasons of the Baltic Sea indicate a clear requirement for bio-optical models and remote sensing methods to adopt optical seasonality. Chla appears the most seasonally variant indicator of the standing stock of phytoplankton in the Baltic Sea. The optical signature of the pigment is severely masked by CDOM, and particulate scattering is too weak to bring out the pigment absorption signature in the apparent optical properties (K_d_, R_rs_) in all but peak bloom conditions. From a purely statistical perspective, TSM and POC are seasonally more robust indicators of the phytoplankton distribution in the pelagic Baltic Sea, compared to Chla. It is interesting that for the Baltic Sea, the use of a single SIOP for phytoplankton using POC as currency may represent the phytoplankton community better across seasons than Chl-a would, and this result may interest e.g. biogeochemical modellers. This does, however, not imply that Chla should be abandoned as an indicator of phytoplankton distributions, particularly since it is most commonly measured in situ. Instead, the sensitivity of optical remote sensing approaches to the seasonally changing role of Chla in the phytoplankton community should be examined, and methods should be developed to optimize separation of absorption by CDOM and particulate matter. Existing Chla retrieval algorithms should also be tested for their sensitivity to the in situ Chla concentration, rather than a correlative response to TSM, as the latter would render them sensitive to overestimation in near-coastal regions and river plumes. The supplementary materials to this paper will support detailed optical-biogeochemical modelling of the Baltic Sea, for which thus far only sparse observations have been made available. Furthermore, the included set of spectral water-leaving reflectance (S1 Appendix and S1 Dataset) supports direct testing of existing and future remote sensing algorithms aiming at retrieval of Chla, TSM, and POC.

## Supporting information

S1 Appendix Description of the simulated Rrs(λ) data (S1 Dataset). (DOCX)

S1 Dataset Simulated Rrs(λ) spectra in spring and summer. These NetCDF-formatted datasets contain Rrs(λ) as a function of solar zenith angle, viewing zenith and azimuth angles, concentrations of chlorophyll-*a*, total suspended matter, and chromophoric dissolved organic matter, and optical properties representive of either spring or summer. Hosted externally under doi: 10.5281/zenodo.254090.
(DOCX)

S1 Table Data distributions. (CSV)

S2 Table Optical-biogeochemical relationships and model fits. (CSV)

S3 Table Spectral diffuse downwelling attenuation coefficient K_d_(λ). (CSV)

S4 Table Absorption coefficient of chromophoric dissolved organic matter *a*_CDOM_(λ). (CSV)

S5 Table Particulate backscattering coefficient. (CSV)

S6 Table Particulate backscattering to scattering ratio β_*p*_(λ). (CSV)

S7 TableMass-specific particulate absorption coefficients $a_{\text{p}}^{*}{{}_{,}}_{\text{chl}}(\lambda )$, $a_{\text{p}}^{*}{{}_{,}}_{\text{TSM}}(\lambda )$, and $a_{\text{p}}^{*}{{}_{,}}_{\text{POC}}(\lambda )$.(CSV)

S8 TableMass-specific pigment absorption coefficients $a_{\phi }^{*}{{}_{,}}_{\text{chl}}(\lambda )$, $a_{\phi }^{*}{{}_{,}}_{\text{TSM}}(\lambda )$, and $a_{\phi }^{*}{{}_{,}}_{\text{POC}}(\lambda )$.(CSV)

S9 TableMass-specific non-algal particle absorption coefficients $a_{\text{nap}}^{*}{{}_{,}}_{\text{chl}}(\lambda )$, $a_{\text{nap}}^{*}{{}_{,}}_{\text{TSM}}(\lambda )$, and $a_{\text{nap}}^{*}{{}_{,}}_{\text{POC}}(\lambda )$.(CSV)

S10 TableMass-specific particulate scattering coefficients $b_{\text{p}}^{*}{{}_{,}}_{\text{chl}}(\lambda )$, $b_{\text{p}}^{*}{{}_{,}}_{\text{TSM}}(\lambda )$, and $b_{\text{p}}^{*}{{}_{,}}_{\text{POC}}(\lambda )$.(CSV)

S11 TableSpectral models of the pigment packaging effect for $a_{\text{p}}^{*}{{}_{,}}_{\text{chl}}(\lambda )$ and $a_{\phi }^{*}{{}_{,}}_{\text{chl}}(\lambda )$.(CSV)
