# Supplementary material for: Contrasting seasonality in optical-biogeochemical properties of the Baltic Sea
Source: PLoS One. 2017 Apr 6;12(4):e0173357. doi: 10.1371/journal.pone.0173357 (PMC5383033; doi:10.1371/journal.pone.0173357)
Supplement: S1 Appendix — (DOCX) [file pone.0173357.s001.docx]

## S1 Appendix

## Forward-modelled reflectance from spring and summer Baltic Sea specific inherent optical properties

Optical models resulting from the analyses presented in the paper are provided in detail in the *supplementary data* (S1-11 Tables) including detailed value distributions and statistics. An extensive dataset of remote-sensing reflectance (R_rs_, units sr^-1^) spectra is additionally provided here (S1 Dataset) based on forward modelling of mean concentration-specific inherent optical properties (SIOPs) for both spring and summer, modelled for a wide range of Chlorophyll-a (Chla), Coloured Dissolved Organic Matter (CDOM), and Total Suspended Matter (TSM) concentrations as well as solar and viewing angles. The primary aim of providing this supplementary dataset is to aid evaluation of remote sensing algorithms for the Baltic Sea in future studies.

**Methods**

All simulations were carried out using the radiative transfer simulation software Hydrolight (version 5.2.0, [1,2]). The built-in four-component model was used as follows: the sum of absorption contained pure water (*a*_w_), phytoplankton pigment *a*_φ_(λ) modelled after Chla-specific *a^*^*_φ,Chl_(λ), ‘non-algal’ particle absorption *a*_nap_(λ), i.e. the difference between total particulate and phytoplankton pigment absorption, modelled after TSM dry-weight specific *a^*^*_nap,TSM_(λ), and *a*_CDOM_ modelled after *a*_CDOM_(412) and season-specific central values of *S*, the spectral slope of *a*_CDOM_(λ). The SIOPs used in the simulations were the mean season-specific spectra described in the main paper and supplementary materials. A freshwater model (salinity = 0) for *a*_w_(λ) was adopted from [3] over the 380-725 nm range. Temperature affects the absorption by water in the UV and NIR, where the constants from [2] measured at 22°C were used. The model for *b*_w_(λ) was that of [4] , also applied with salinity = 0, and the scattering phase function of pure water was adopted from [5]. Particulate scattering *b*_p_(λ) was modelled after TSM dry-weight specific *b*^*^_p_,_TSM_(λ) which combines all particulate matter (i.e. no separate definitions for pigmented and non-pigmented material). The backscattering-to-scattering ratios for spring (0.018) and summer (0.014) were adopted from the spectral means shown in the main text of this paper. The scattering phase function of particles followed the Fournier–Forand model implemented in Hydrolight.

Concentrations used in forward simulations of R_rs_. Concentrations closest to the first percentile (P1), median (P50), and 99^th^ percentile (P99) of concentration ranges observed in situ are indicated in their respective bold-print columns. First and last columns list concentrations for which forward simulations should be considered out-of-scope (OOS) of this data set.

|  | OOS |  | P1 |  |  |  |  | P50 |  |  |  | P99 |  | OOS |
| --- | --- | --- | --- | --- | --- | --- | --- | --- | --- | --- | --- | --- | --- | --- |
| Spring |  |  |  |  |  |  |  |  |  |  |  |  |  |  |
| Chla (mg m^-3^) | 0.10 | 1.0 | **2.0** | 4.0 | 6.0 | 8.0 | 10.0 | **14.0** | 20.0 | 26.0 | 32.0 | **42.0** | 84.0 | 250.0 |
| TSM(g m^-3^) | 0.05 | 0.4 | **0.8** | 1.3 |  | 1.8 |  | **2.3** | 4.0 | 5.7 | 7.4 | **8.9** | 18.0 | 50.0 |
| a_CDOM_412(m^-1^) | 0.05 | 0.2 | **0.3** |  | 0.5 |  |  | **0.7** | 0.9 | 1.2 | 1.5 | **3.0** |  | 20.0 |
| Summer |  |  |  |  |  |  |  |  |  |  |  |  |  |  |
| Chla (mg m^-3^) | 0.10 | 0.8 | **1.7** | 2.5 |  | 3.3 |  | **4.2** | 6.2 | 8.2 | 10.2 | **12.8** | 26.0 | 120.0 |
| TSM(g m^-3^) | 0.05 | 0.2 | **0.3** | 0.8 |  | 1.3 |  | **1.8** | 3.4 | 5.0 | 6.6 | **8.1** | 16.0 | 50.0 |
| a_CDOM_412(m^-1^) | 0.05 | 0.2 | **0.3** |  |  | 0.5 |  | **0.7** | 0.9 | 1.2 | 1.5 | **3.0** |  | 20.0 |

The range of concentrations of Chla, TSM, and *a*_CDOM_(412) for which forward modelled R_rs_ data are provided is described in the table included here. By adopting mean concentration-specific IOPs it is assumed that the measured SIOPs are valid over the whole concentration range and all combinations of optically active constituents for which they were modelled. This simplification was considered acceptable for the purpose of evaluating algorithm performance but should not be considered strictly representative of variations in SIOPs that will occur in nature over such a wide concentration range. Simulated concentrations ranged from the first percentile (0.5 × P1) to the 99^th^ percentile observed in the *in situ* data described in the main paper. In addition, the simulations included one or two values beyond this range, as well as an extreme case at the low and high bound, which can be used to catch out-of-scope solutions in iterative comparisons of forward and inverse modelled R_rs_. All combinations of the component concentrations resulted in 14 Chla, 12 TSM, and 10 *a*_CDOM_(412) steps to model the spring season (1680 combinations), and 12 Chla, 12, TSM, and 10 *a*_CDOM_(412) steps for the summer season (1440 combinations). Dependence of *a^*^*_φ,Chl_(λ) on Chla concentration (the ‘pigment package effect’) was included in the forward simulations, following *a^*^*_φ,Chl_(λ) = A(λ) Chla^B(λ)^ as suggested in [6]. The empirical coefficients A(λ) and B(λ) are described in and provided as supplementary data to the main paper.

The RADTRAN-X based semi-empirical sky model implemented in Hydrolight was used with atmospheric parameters set to atmospheric pressure 101.3 kPa, air mass type 5, relative humidity 80%, precipitable water 2.0 cm, visibility 40 km, and ozone 400 Dobson units in spring and 300 Dobson units in summer. The index of refraction of water was set to 1.34 and wind speed to 5.0 m s^−1^. Simulations were made for five sun zenith angles (30°–70°, at 10° steps), at six viewing zenith angles (0°–50°, at 10° steps) and 13 azimuth angles (0°–180°, at 15° steps). Thus, the combined in-water and geometric permutations resulted in 655200 R_rs_ spectra for spring and 561600 for summer scenarios.

Simulations covered the 351.25–901.25 nm wavelength range with 2.5 nm bandwidth (220 bands). This range allows downstream simulation of both optical satellite sensors and commonly used *in situ* spectroradiometers. The simulated library consisting of input concentrations, solar and viewing angles, and R_rs_ spectra) is stored in NetCDF4 format as supplementary S1 Dataset. The NetCDF4 format is self-describing and interface libraries exist for most programming environments. Examples of how to extract information on the included dimensions and variables, and of how to plot a series of R_rs_ spectra in Python using the *netCDF4* and *matplotlib* libraries is provided below.

**References**

1. Mobley CD, Sundman LK (2013) Hydrolight 5.2 Users’ quide. Sequoia Scientific, Inc.: 104.

2. Mobley CD, Sundman LK (2013) Hydrolight 5.2 Technical documentation. Sequoia Scientific, Inc.: 110.

3. Pope RM, Fry ES (1997) Absorption spectrum 380–700 nm of pure water. II. Integrating cavity measurements. Appl Opt 36: 8710–8723.

4. Morel A (1974) Optical properties of pure water and pure sea water. In: Jerlov MG, Nielsen ES, editors. Optical Aspects of Oceanography. New York: Academic Press. pp. 1–24.

5. Mobley CD (1994) Light and Water: Radiative Transfer in Natural Waters. San Diego, California: Academic Press.

6. Bricaud A, Babin M, Morel A, Claustre H (1995) Variability in the chlorophyll-specific absorption-coefficients of natural phytoplankton - analysis and parameterization. Journal of Geophysical Research-Oceans 100: 13321-13332.

**Code snippets (for Python)**

The following is a Python script containing code snippets demonstrating how to use the *netCDF4* and *matlotlib* libraries to read and plot the simulated Rrs(λ) dataset (S1 Dataset) included with this paper. Please note that the file path of the netcdf file to be inspected must be specified in line 11.

1. # -*- coding: utf-8 -*-
2. """
3. Code snippets demonstrating the use of the Baltic Sea 
   simulated reflectance data set in Python
4. """
5. # import libraries (assuming they are installed)
6. **import** netCDF4 as nc
7. **import** matplotlib.pyplot as plt
8. # revise the path below to reflect the location of the spring or summer netcdf file
9. path_to_netcdf = r'.\HL_BS_Spring_v1_0_0.nc'
10. # create a file handle
11. nc_handle = nc.Dataset(path_to_netcdf, 'r')
12. # to show dataset information:
13. **print**(nc_handle)
14. # to list the data dimensions and their sizes:
15. **for** dim **in** nc_handle.dimensions.keys():
16. dimname = nc_handle.dimensions[dim].name
17. dimsize = len(nc_handle.dimensions[dim])
18. **print**("{name}: size {size}".format(name=dimname, size=dimsize))
19. # to list each variables and its attributes:
20. **for** var **in** nc_handle.variables:
21. **for** attr **in** nc_handle.variables[var].ncattrs():
22. value = nc_handle.variables[var].getncattr(attr)
23. **print**("{varname}, {attribute}={value}".format(varname=var,
24. attribute=attr,
25. value=value))
26. vardims = nc_handle.variables[var].dimensions
27. **print**("{varname}, dimensions={vardims}".format(varname=var,
28. vardims=vardims))
29. # variable names that are also dimension names describe the spacing of the Rrs
30. # data along that dimension. Extract these as arrays, e.g. for plotting purposes:
31. sun_zenith = nc_handle.variables[u'sun_zenith'][:]
32. view_theta = nc_handle.variables[u'view_theta'][:]
33. acdom = nc_handle.variables[u'acdom'][:]
34. chla = nc_handle.variables[u'chla'][:]
35. tsm = nc_handle.variables[u'tsm'][:]
36. view_phi = nc_handle.variables[u'view_phi'][:]
37. wavelength = nc_handle.variables[u'wavelength'][:]
38. # The Rrs data are given along the above 7 dimensions.
39. # To show the order in which the dimensions are used:
40. **print**(nc_handle.variables['Rrs'].dimensions)
41. # Select a slice of Rrs data, e.g. along the following conditions:
42. sun_zenith_index = 0
43. view_theta_index = 9
44. acdom_index = 2
45. chla_index = 4
46. tsm_index = 4
47. view_phi_index = 6
48. # Print the selection
49. **print**("Sun zenith:\t\t{sunz}\n"
50. "Viewing theta:\t{the}\n"
51. "aCDOM:\t\t{acd}\n"
52. "Chl-a:\t\t{chl}\n"
53. "TSM:\t\t{tsm}\n"
54. "Viewing phi:\t{phi}".format(sunz=sun_zenith[sun_zenith_index],
55. the=view_theta[view_theta_index],
56. acd=acdom[acdom_index],
57. chl=chla[chla_index],
58. tsm=tsm[tsm_index],
59. phi=view_phi[view_phi_index]))
60. # Create a handle to the Rrs data (for convenience)
61. Rrs = nc_handle.variables['Rrs']
62. # Select the slice. The index for wavelength includes all (extract full spectrum)
63. Rrs_slice = Rrs[sun_zenith_index, view_theta_index, acdom_index,
64. chla_index, tsm_index, view_phi_index, :]
65. # to plot this spectrum using matplotlib:
66. plt.clf()
67. plt.plot(wavelength, Rrs_slice, 'k')
68. plt.show()
69. # Or obtain a larger slice, for example all spectra along the chla dimension:
70. Rrs_slice = Rrs[sun_zenith_index, view_theta_index, acdom_index,
71. :, tsm_index, view_phi_index, :]
72. **print**(Rrs_slice.shape)
73. plt.clf()
74. **for** R **in** range(len(chla)):
75. plt.plot(wavelength, Rrs_slice[R,:])
76. plt.legend(chla)
77. plt.show()
78. # Similarly we can plot the effect of sun zenith angle on Rrs:
79. Rrs_slice = Rrs[:, view_theta_index, acdom_index,
80. chla_index, tsm_index, view_phi_index, :]
81. **print**(Rrs_slice.shape)
82. plt.clf()
83. **for** R **in** range(len(sun_zenith)):
84. plt.plot(wavelength, Rrs_slice[R,:])
85. plt.legend(sun_zenith)
86. plt.show()
87. # close file
88. nc_handle.close()
