# Supplementary material for: Contrasting seasonality in optical-biogeochemical properties of the Baltic Sea
Source: PLoS One. 2017 Apr 6;12(4):e0173357. doi: 10.1371/journal.pone.0173357 (PMC5383033; doi:10.1371/journal.pone.0173357)
Supplement: S1 Dataset — These NetCDF-formatted datasets contain Rrs(λ) as a function of solar zenith angle, viewing zenith and azimuth angles, concentrations of chlorophyll-a, total suspended matter, and chromophoric dissolved organic matter, and optical properties representive of either spring or summer. Hosted externally under doi: 10.5281/zenodo.254090. (DOCX) [file pone.0173357.s002.docx]

**S1 Dataset. Simulated Rrs(λ) spectra in spring and summer.** These NetCDF-formatted datasets contain Rrs(λ) as a function of solar zenith angle, viewing zenith and azimuth angles, concentrations of chlorophyll-*a*, total suspended matter, and chromophoric dissolved organic matter, and optical properties representive of either spring or summer. Hosted externally under doi: 10.5281/zenodo.254090.
